# Risk factors for *Neospora caninum*, bovine viral diarrhoea virus, and *Leptospira interrogans* serovar Hardjo infection in smallholder cattle and buffalo in Lao PDR

**DOI:** 10.1371/journal.pone.0220335

**Published:** 2019-08-08

**Authors:** Luisa Olmo, Michael P. Reichel, Sonevilay Nampanya, Syseng Khounsy, Lloyd C. Wahl, Bethanie A. Clark, Peter C. Thomson, Peter A. Windsor, Russell D. Bush

**Affiliations:** 1 Sydney School of Veterinary Science, The University of Sydney, Camden, NSW, Australia; 2 Jockey Club College of Veterinary Medicine and Life Sciences, City University of Hong Kong, Tat Chee Avenue, Kowloon, Hong Kong SAR, China; 3 Department of Livestock and Fisheries, Ministry of Agriculture and Forestry, Vientiane, Lao PDR; 4 School of Life and Environmental Sciences, The University of Sydney, Camden, NSW, Australia; University of Kentucky College of Medicine, UNITED STATES

## Abstract

Smallholder large ruminant production in Lao People’s Democratic Republic (Laos) is characterised by low reproductive efficiency. To determine if common abortifacient bovid infectious diseases are involved, a serological investigation was conducted. Sera was collected from stored and fresh cattle (*n =* 390) and buffalo (*n* = 130) samples from 2016–18 from, and then examined for associations in a retrospective risk factor study of 71 herds. The sera were assayed for antibodies to *Neospora caninum*, bovine viral diarrhoea virus (BVDV), *Leptospira interrogans* serovar Hardjo and *Brucella abortus* using commercially available enzyme-linked immunosorbent assay kits. These pathogens were detected in buffalo samples at 78.5% (95% CI 71.4–85.6), 0%, 2.3% (95% CI 0–4.9) and 0%, respectively, and in cattle at 4.4% (95% CI 2.4–6.4), 7.7% (95% CI 3.1–12.3), 12.8% (95% CI 9.5–16.1) and 0.26% (95% CI 0–0.8), respectively. Exposure of buffalo to *N*. *caninum* was positively associated with buffalo age, with a predicted seropositivity at birth of 52.8%, increasing to 97.2% by 12 years of age (*p* = 0.037). Exposure of cattle to *L*. *interrogans* serovar Hardjo was more prevalent in females compared to males, was associated with higher titres of BVDV, and was more prevalent in the wet season compared to the dry season. Exposure of cattle to BVDV was more prevalent in males compared to females, the wet and dry seasons were comparable, and was associated with rising antibody titres against *N*. *caninum* and *L*. *interrogans* serovar Hardjo. The risk factor survey identified that the probability of herds being *N*. *caninum* positive increased with farmer age, if farmers believed there were rodents on farm, and if farmers weren’t aware that canids or rodents could contaminate bovid feed on their farm. The probability of a herd being positive to *L*. *interrogans* serovar Hardjo increased on farms where multiple cows shared the same bull, where farmers had lower husbandry knowledge, and on farms that used water troughs. The probability of a herd being BVDV seropositive increased with increasing herd size and increasing titres to *N*. *caninum*. The benchmarking of bovid exposure to emerging abortifacient pathogens and identification of their risk factors potentially informs disease prevention strategies, supporting efforts to establish a biosecure beef supply for enhanced smallholder livestock productivity, public health and food security in Laos and surrounding countries.

## Introduction

Lao People’s Democratic Republic (Laos) is a low-middle income country of approximately 7 million people located in South-east Asia [1]. Agriculture is the main source of employment and is essential to the livelihoods of approximately 70% of the population [2]. Farms are predominantly smallholder, existing on mean plots of 2.1 ha which is mainly dedicated to cultivating rain-fed rice [2]. Herds of 5–10 cattle and buffalo are integral to these holdings, traditionally used for draught power for ploughing paddy fields, but more recently as a household bank and increasingly as beef [3]. From 2000 to 2012, cattle and buffalo live weight prices (USD/tonne) increased rapidly by more than 500% and 800%, respectively [1]. With rising prices, interest in improving large ruminant productivity has been increasing and more recently, enhancing reproductive efficiency is achieving recognition as a valuable opportunity to raise smallholder incomes that may assist alleviation of rural poverty. Of concern, village biosecurity is generally lacking and the increasing regional livestock trade remains poorly regulated, with ‘informal’ international trade persisting [4]. As Laos is an important livestock trade thoroughfare in South-east Asia, the risk of emerging and transboundary infectious diseases, and particularly foot-and-mouth disease (FMD), increases with the growth of regional livestock trade, threatening attempts to progress livestock productivity [3].

Regionally prevalent large-ruminant pathogens of reproductive importance include *Neospora caninum*, bovine viral diarrhoea virus (BVDV), *Leptospira interrogans* and *Brucella abortus*. All potentially cause lowered fertility, still birth, abortion and congenital malformations [5–8], and serological evidence of the first 3 of these pathogens was recently identified in Laos [9].

Cattle and buffalo are indirect hosts of the apicomplexan protozoan parasite *N*. *caninum* [5]. Livestock become infected by ingesting food and water contaminated by oocysts shed in faeces of the definitive canine host [10], while canids can become infected from consumption of bovid tissue containing bradyzoites [5]. Cows can transfer infection to offspring *in utero* resulting in reproductive failure [11] that has been estimated to have a global annual cost of USD 1.3 billion [12]. Exposure of buffalo to *N*. *caninum* at 68.9% (*n* = 61) was identified on preliminary screening in Laos and was significantly higher than co-reared cattle at 7.8% (*n* = 90) [9]. The high susceptibility of buffalo to *N*. *caninum* infection has been documented globally [13, 14]. However, there is uncertainty if this represents inherent species susceptibility or species management issues or both [13]. Regardless, an understanding of the role of species-specific management, including whether buffalos being reared closer to the homestead increases their exposure to the faeces of village dogs [15], is required if village-level infection preventative strategies are to be applied in Laos and beyond. Currently, with all 4 reproductive pathogens of interest, infection control by vaccination is not available in Laos due to both a lack of local availability [15] and absence of farmer and animal health worker awareness of the presence of these pathogens. Therefore, an understanding of the risk factors of these pathogens that is specific to Lao smallholders is critical in developing knowledge-based infection preventative strategies involving management interventions, that can be disseminated locally by government extension staff.

Bovine viral diarrhoea virus (BVDV) is a pestivirus of the *Flaviviridae* family that is transmitted through direct or venereal contact with an infected bovid [8]. Cows exposed during gestation can infect offspring *in utero* resulting in reproductive loss, congenital malformations or the birth of persistently infected (PI), immunotolerant calves that shed the virus for their lifetime [16]. The low BVDV exposure rates in Laos of 4.9% in buffalo and 10% in cattle [9] suggest that PI calves are not common. This may reflect the ability of small herds to break the viral cycle because acute infection is self-cleared before it is transmitted [17, 18]. However, as herd sizes and trade from endemic countries continues to increase, the risk of exposure to PI animals also increases [2, 19]. Assessing the role of potential risk factors, including the trade of pregnant animals [20], the sharing of bulls [21] common grazing [15] and the low levels of biosecurity, will be needed to develop infection and presumably, disease prevention strategies.

Leptospires are bacteria shed in urine that can infect large ruminants through abrasions. They colonise the renal tubes, mammary glands, and reproductive tracts [22] leading to infertility and abortion [23]. Leptospires survive well in warm/humid conditions [24] and water-borne transmission may facilitate spread to naïve herds in flood-prone regions common in the tropical countries. In Laos, antibodies against *Leptospira* were detected in 6.0% and 1.7% of cattle and buffalo, respectively, in 2012 [25] and *Leptospira interrogans* serovar Hardjo was detected at 22.2% and 3.3% in 2017, respectively. As exposure has appeared to have increased from 2012–2017 and seroprevalence differed significantly between regions [9], a better understanding of factors conducive to transmission are needed to limit spread of the infections and potential for disease in Laos.

Leptospirosis is an important zoonotic disease and *Leptospira* antibodies were detected in 23.9% of participants in a human sero-survey from central Laos in 2008 [26]. As smallholder households are at high occupational risk of infection, usually residing in close proximity to their livestock and working in flooded rice fields fertilised by bovine manure [27], research is needed to develop strategies to limit the presence of infection in livestock to reduce human health risks.

Another abortifacient and zoonotic pathogen of interest in Laos is *Brucella abortus*, typically infecting cattle through ingestion of bacteria from foetal fluids and placental membranes [28]. Humans are usually accidental hosts, becoming infected from consuming unpasteurised milk, from handling infected animals [6], or from accidental self-administration of live (Strain 19) vaccines, resulting in a range of acute (Undulant Fever) to chronic illnesses, or miscarriage in pregnant women [29]. Three studies conducted from 2012–2017 have established that antibodies to *Brucella* spp. are absent in Lao bovids [9, 25, 30]. However, the risk of incursion remains high due to unofficial livestock importations from endemic countries [4], particularly to establish intensive dairy enterprises. As dairy consumption is increasing rapidly in Laos [31], monitoring for the introduction of *B*. *abortus* is of increasing concern for human health.

Bovid abortion has not been previously quantified in Laos, although low reproductive efficiency observed [3] and anecdotal reports of bovid abortion [9] suggest it is a potentially important yet a largely unrecognised constraint to livestock production. The high rates of exposure to abortifacient pathogens suggests the likelihood that unreported reproductive disease contributes to reproductive loss and the observed low levels of bovine reproductive efficiency. Numerous studies have shown that positive sero-status for *N*. *caninum* in cattle and buffalo is generally linked to increased rates of abortion [7, 32–34]. To assist in developing species-specific prevention strategies for infectious causes of abortion in Laos, we assessed the seroprevalence of these pathogens in a larger sample of bovids than the preliminary screening [9] and identified potential risk factors associated with exposure.

## Materials and methods

### Ethics statement

The methodologies used in this study complied with the National Health and Medical Research Council’s (NHMRC) National Statement on Ethical Conduct in Human Research (2007) and the Universities Australia Australian Code for the Responsible Conduct of Research. Animal and human ethics approval was obtained from the University of Sydney Ethics Committee (project no. 2015/765 and 2014/783, respectively). This included approval of the verbal consent procedure where collection of animal blood samples and farmer interviews only proceeded if farmers verbally consented which was recorded on a list. Written consent was not possible due to many farmers being illiterate in Lao language due to varying ethnolinguistic groups and education.

### Serum sample collection

Frozen serum samples were collected from cattle and buffalo > 6 months of age, stored at the National Animal Health Laboratory (NAHL), Vientiane (*n =* 473). These frozen samples were originally derived from the ‘NZ OIE DLF FMD Control Project’ (OIE-DLF) [35] and were collected from October-November of 2016 (*n* = 235), and a University of Sydney project entitled; ‘Enhancing transboundary livestock disease risk management in Lao PDR’ (ETLDRM) [36] collected from May-June of 2017. Samples were taken from 5 agriculturally-significant provinces of Bokeo (BK), Luang Prabang (LPB), Vientiane (VTE), Xieng Khoung (XK), and Xayabouli (XB). A summary of sample origin is presented (Table 1) demonstrating that buffalo samples were not available from VTE and BK, partially due to the declining national buffalo population. To approach the sample size calculated from online epidemiological tools, an additional 47 fresh samples were collected from a buffalo dairy and associated villages in Luang Prabang in February 2018. The collection of another 50 buffalo samples were intended but did not eventuate due to limited numbers of buffalo on participating farms and advice from government authorities not to excessively inconvenience farmers. Data were recorded for each serum sample and included: sex, species (cattle/buffalo), farm and village species (cattle, buffalo or both), source of data (ETLDRM, OIE-DLF, buffalo dairy), province, district, village, season (wet/dry), animal age group (≤ 1, 2–3, 4–5, ≥ 6 years old), and body condition score (BCS; thin, medium and fat). Samples collected from the dairy and associated villages were by jugular venepuncture in February 2018. These samples were centrifuged on the same day as collection for 5 minutes, transferred to transport tubes and stored at -80°C until international transfer. Samples taken from frozen sera stored at the NAHL were defrosted and transferred to transport tubes. Sera were transported from Laos to the Veterinary Diagnostic Laboratory (VDL) at the City University of Hong Kong, stored in chilled BioTherm IATA-Standard specimen containers.

**Table 1:** Summary of cattle and buffalo serum sample locations from a serological study conducted in Lao PDR in 2018.

Province	District	Village	*n cattle*	*n buffalo*	*N*
**OIE-DLF samples**					
Bokeo	Houay Xay	Don Pau	20	-	
	Pak Tha	Huy Khot	16	-	
		Hoy Sak	20	-	56
Luang Prabang	Luang Prabang	Long Lun	-	20	
	Pak Ou	Somsanouk	19	1	
		Hardkor	19	1	60
Vientiane	Vang Veing	Phatang	20	-	
		Nathong	20	-	
		Namouang	20	-	60
Xieng Khoung	Bpae	Nam Ka	16	4	
	Pek	Tha	14	6	
		Bua Kob	20	-	60
Xayabouli	Hongsa	Phonxay	15	5	
		Siboun Huan	20	-	
	Phiang	Nong Ngoua	16	3	59
**ETLDRM samples**					
Luang Prabang,	Pak Ou	Hardkor	3	17	
		Hardkam	4	16	
		Phonhom	20	-	60
Xieng Khoung	Phou Kout	Naxaythong	18	2	
		Laethong	16	4	
		Bong	15	4	59
Xayabouli	Phiang	Naboum	20	-	
		Phonsavang	19	-	
		Nong Houng	20	-	59
**Buffalo dairy**					
Luang Prabang	Luang Prabang	Khok Man	-	4	
		Luang Prabang	-	1	
		Thinkeo	-	14	
		Phabat	-	2	
		MK	-	3	
		PikYai	-	18	
		College	-	1	
		Maung Khav	-	4	47
			**Total**		520

*n*: no. of samples; *N*: total no. of samples; ETLDRM: Enhancing transboundary livestock disease risk management in Lao PDR project; OIE-DLF: NZ OIE DLF FMD Control Project

### Serological analysis

Commercially-available enzyme-linked immunosorbent assay (ELISA) kits were used in accordance with the manufacturer’s instructions and cut-off recommendations (Table 2). An exception was made for the *N*. *caninum* IDEXX ELISA with a lower sample to positive (S/P) ratio cut-off of 21% (manufacturer’s recommended S/P ratio cut-off of 50%) to maximise the diagnostic sensitivity of an approximate equivalence to an indirect fluorescent antibody test (IFAT) titre of 1:200 [37].

**Table 2:** Enzyme-linked immunosorbent assays (ELISA) used for the determination of antibodies against *Neospora caninum*, bovine viral diarrhoea virus (BVDV), *Leptospira interrogans* serovar Hardjo (*L*. *hardjo*) and *Brucella abortus* in cattle and buffalo sera from Lao PDR.

Pathogen	ELISA	OD (nm)	Calculating test	Test cut-off (%)
*N*. *caninum*	IDEXX Neospora X2 ^a^	620	$S/P=\frac{SampleA_{620}-NC\bar{x}}{PC\bar{x}-NC\bar{x}}$	≥ 21
BVDV	IDEXX BVDV Total Antibody Test Kit ^b^	450	$S/P=\frac{SampleA_{450}-NC\bar{x}}{PC\bar{x}-NC\bar{x}}$	≥ 30
*L*. *hardjo*	PrioCHECK L. hardjo Ab Test ^c^	450	$PP=\frac{corOD_{450}testsample}{corOD_{450}ReferenceSerum1}$	> 45
*B*. *abortus*	IDEXX Brucellosis Antibody Test Kit ^d^	450	$S/P=\frac{SampleA_{450}-NC\bar{x}}{PC\bar{x}-NC\bar{x}}$	> 120

S/P: sample to positive ratio; PP: percentage positivity; nm: nanometres; $NC\overset{¯}{x}$: negative control mean; $PC\overset{¯}{x}$: positive control mean; OD: optical density; cor: corrected

^a^ IDEXX *Neospora* X2 Ab test, *Neospora caninum* antibody test kit, IDEXX Laboratories, Westbrook, Maine, USA.

^b^ IDEXX BVDV Total Ab test, Bovine Viral Diarrhoea Virus (BVDV) Antibody Test Kit, IDEXX Switzerland AG, Liebefeld-Bern, Switzerland

^c^ PrioCHECK L. hardjo Ab Test, Prionics AG, Schlieren-Zurich, Switzerland

^d^ IDEXX Brucellosis Serum, Brucellosis Antibody Test Kit, IDEXX Laboratories, Westbrook, Maine, USA

### Epidemiological survey and design

Face-to-face interviews were conducted on a subset of 71 of the 211 households involved in the blood collection. The survey sample size achieved was a compromise of what local authorities deemed appropriate based on resource prioritisation and the logistical constraints of travelling to all villages. All 5 provinces were surveyed, with 1 district selected per province and 2 villages selected per district (*n* = 10). Villages were selected purposefully to ensure variability in village-level seroprevalence for all pathogens. Households were selected in each village based on their involvement in the blood collection, willingness to participate and availability. On average most farmers had 2 large ruminants sampled. Interviews were pre-arranged by staff from the Department of Livestock and Fisheries, Vientiane (DLF), the Luang Prabang Provincial Agricultural and Forestry Office (PAFO) and respective District Agricultural Forestry Offices (DAFO), with permissions received from local authorities including village chiefs. The survey was drafted in English based on a literature review of known risk factors, then translated and conducted in Lao language by DLF staff with animal health knowledge. The survey was pre-tested with 4 farmers by PAFO staff and modified to improve farmer responsiveness. Each farmer survey took approximately 20 minutes to complete. Most surveys were conducted by PAFO staff (49/75) with the remainder conducted by DAFO staff following their receipt of training on the day prior to interviews. Farmers were briefed on the purpose and importance of providing accurate answers, prior to the conduct of the surveys.

Due to delays between sample and survey collection, individual animal reproductive history was not included as Lao smallholder farmers do not keep any written records and there was a risk that such information if provided, would be inaccurate because of recall bias. Instead, the survey focused on household level practices and herd history of reproductive problems (S1 and S2 Texts).

### Statistical analysis

#### Animal-level serology and risk factors

Animal-level seroprevalence was interpreted as binary outcomes ‘0’ for negative or inconclusive results and ‘1’ for positive results, with seroprevalence determined for each pathogen and livestock species. Assessment of risk factors was conducted when the seroprevalence for the pathogen × livestock species combination exceeded 5% (buffalo *N*. *caninum*, cattle *L*. *interrogans* serovar Hardjo and cattle BVDV). Logistic generalised linear mixed models (GLMM) were fitted to the binary seroprevalence data. The available explanatory variables were codes for date of serum collection, season, year, province, district, village, herd and village species, sex, age, BCS, and source of serum. Additionally, BVDV sero-status, antibody titre optical density (OD) and S/P ratio were included as explanatory variables for *N*. *caninum* and *L*. *interrogans* serovar Hardjo binary outcome variables and sero-status, antibody titre OD and S/P ratio or percentage positivity (PP) for *N*. *caninum* and *L*. *interrogans* serovar Hardjo were used as explanatory variables for the BVDV binary outcome variable because of BVDVs immunosuppressive properties [38]. Variables with noticeable skewness were logarithmically transformed, to reduce the effect of influential values. Transformations were retained if improvements were observed in histograms. Collinearity was addressed by deriving the correlation coefficients (*r*) between numeric variables. In cases where 2 variables had *r >* 0.65, the variable with the weaker correlation to the outcome variable was removed. Variables were then submitted for univariable analysis where province, district, village and herd were included as random effects. Variables with *p <* 0.2 in univariable analysis were included in the initial multivariable model which underwent backward elimination until all variables had a *p-value* of < 0.1. In the final model, variables with *p* < 0.1 were considered suggestive of associations and variables with *p* < 0.05 were considered significantly associated with the outcome variable. Splines were fitted to significant predictors with possible nonlinear relationships to explanatory variables and were retained if there were demonstrable improvements in the predicted probability plots. Model fitting and model-based predictions were conducted using the ‘asreml’ function in the asreml package R [39]. Fisher exact tests were also used to provide basic assessments of variation in seroprevalence between provinces.

#### Herd-level serology and risk factors

Surveys were entered and cleaned in Microsoft Excel (2016). Descriptive statistics were used to determine trends and to summarise management practices. To assess the degree of household engagement in risk factors, 3 risk practice scores were calculated for each detected pathogen based on a methodology previously used to assess brucellosis in Pakistan [40]. The Neosporosis risk score (from 0–5), the BVDV risk score (from 0–4) and the Leptospirosis risk score (from 0–5) were the total number of risky practices performed by households specific to each pathogen (Table 3). These were determined through reviewing literature on known risk factors, selecting factors relevant to the Lao smallholder context and only including variables with sufficient variability (>5% variability in responses). The scores were used as ordinal scale variables where explanatory variables were assessed for their effect on these scores using ordinal logistic regression. The explanatory variables considered were demographic factors; farmer sex, age (≤ 40, 41–45, 46–50, 51–55, 56–60, >60 years), education level (no formal education, primary, secondary, tertiary), years of farming experience (1–5, 6–10, 11–15, 16–20, >20), and distance to nearest town (<5, 6–10, 11–15, 16–20, >20 km). Knowledge variables included knowledge that abortion in large ruminants could be caused by disease (No, I don’t know, Yes), knowledge that large ruminant diseases can infect humans (No, I don’t know, Yes), and knowledge that large ruminants could get diseases from dogs or rodents (No, I don’t know, Yes). Farm characteristics included farm species (cattle, buffalo or both), farm land size (ha: <1, 1 to <2, 2 to <3, 3 to <4, ≥4), number of females > 6 months old (≤ 5, 6–10, >10) and history of herd reproductive problems (Yes/No). The same process of variable filtering and model fitting was used as per the GLMMs except that model fitting and model-based predictions were conducted using the ‘clmm’ function in the ordinal package [41], the ‘emmeans’ function in the emmeans package [42] and the ‘rating.emmeans’ function in the RVAideMemoire package [43] in R.

**Table 3:** Farm practices which pose risk of contracting reproductive pathogens in large ruminants.

*Neospora caninum*	Bovine viral diarrhoea virus	*Leptospira interrogans*
Dogs eat aborted foetus/ Placenta/ dead calves	Presence of goats	Presence of rodents
Dogs or rodents defecating near large ruminant feed	Common grazing	Dogs and/or rodents defecate or urinate near bovid feed
Calving animals not isolated from herd	Introduced large ruminants to herd in last 24 months	Allow grazing near flooded rice plots
Borrow equipment from neighbours	Cows share bulls	Presence of pigs
Manure not removed from calving areas weekly		Introduced large ruminants to herd in last 24 months

In addition, 3 logistic GLMM analyses were conducted to assess associations between the demographic factors, all individual disease risk factors, all management practices, and reproductive performance indicators against herd-level sero-status where positive households had at least 1 large ruminant positive to *N*. *caninum*, BVDV, or *L*. *interrogans* serovar Hardjo, respectively.

## Results

### Demographic factors

**Fig 1:**
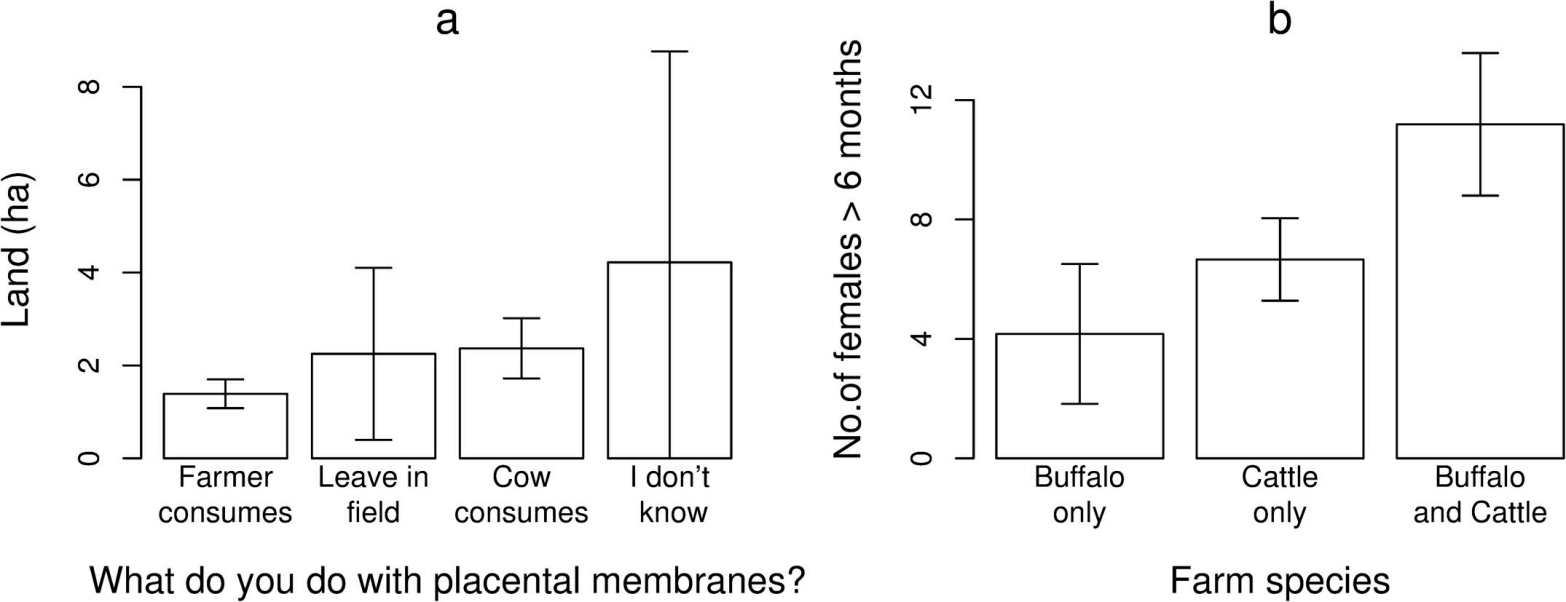
Bar charts conveying trends on smallholder farms in 2018, Lao PDR. (a) The average land size associated with the different disposal methods of large ruminant placental membranes (b) The average number of female large ruminants > 6 months of age associated with farm species. Error bars shown are ± SE.

Of the 75 interviewed farmers, 66 (86.8%) were the primary large ruminant carer in their household. Of these, the majority were male (92.4%), and only 5 were female (7.6%). Respondents were predominantly primary school educated (58.7%), fewer were secondary school educated (32.0%), 2 were tertiary school educated (2.7%) and 5 had no formal education (6.7%). On average, respondents had raised bovids for 15.2 ± 11.5 years (mean ± SD), on 1.9 ± 2.4 ha of land distributed across 2.1 ± 1.2 land parcels. Households were on average 16.7 ± 14.4 km from their nearest town and 17.2 ± 15.4 km from their nearest main road. Farmers raising both cattle and buffalo had an average of 11.2 ± 4.9 females older than 6 months which was larger than herds with only cattle or buffalo (Fig 1B). All cattle and buffalo owned were of the native Laotian yellow and native swamp buffalo breeds, respectively.

### Reproductive practices and performance

**Fig 2:**
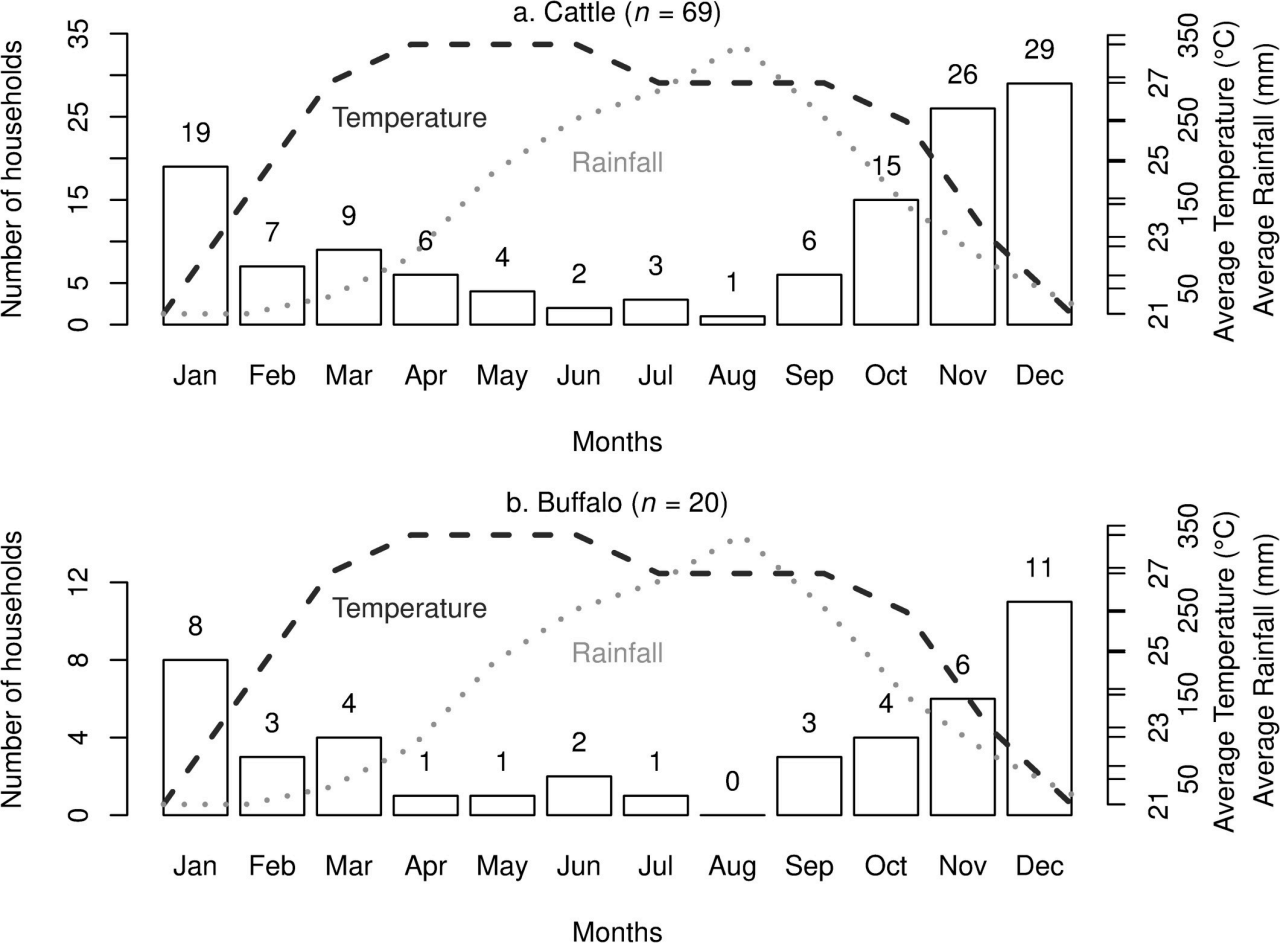
Histograms of the main calving months reported by smallholder farms in 2018, Lao PDR. (a) cattle calves (b) buffalo calves. Temperature data are from the Meteorological Organization Standard Normals 1961–1990 [44] and rainfall data from the Climate Change Knowledge Portal 1901–2015 [45].

A summary of reproductive husbandry practices employed by respondents is presented (Table 4). Almost all famers (97.3%) engaged in unrestricted mating as their main method of breeding bovids. The main methods of pregnancy detection were observing ‘increasing abdomen size’ (49.3%) or ‘increasing udder size’ (33.3%). Only 3 farmers reported using a lack of return to oestrus to detect pregnancy (4.0%). A minority of 14.5% and 10.0% of farmers reported having reproductive problems in their cattle and buffalo herd in the last 24 months, respectively. Farmers typically consumed bovid placental membranes (61.3%) and these farmers typically had < 2 ha of land (Fig 1A). Farmers could only detect pregnancy at 3.9 ± 1.2 months and 5.1 ± 1.9 months gestation in cattle and buffalo, respectively, and reported estimated calving to conception intervals (CCI) of 4.5 ± 3.0 and 8.5 ± 4.3 months, respectively. Calving predominantly occurred from November to January in both cattle and buffalo (Fig 2).

**Table 4:** Reproductive husbandry practices in smallholder cattle and buffalo farms in Lao PDR from an epidemiological survey (*n* = 75).

	Category	*n*	%	95% CI
Main method that your cows get pregnant	Unrestricted mating	73	97.3	93.7–100
	Farmer selects bull from own herd	2	2.7	0–6.3
How do you detect pregnancy?	a) Increased abdomen size	37	49.3	38.0–60.6
	b) Increased udder size	25	33.3	22.6–44.0
	c) Does not return to oestrus	3	4.0	0–8.4
	d) Stops lactating	1	1.3	0–3.9
	e) A mix of a), b) and c)	6	8.0	1.9–14.1
	I don’t know	3	4.0	0–8.4
Have you experienced reproductive problems in the last 24 months? (Infertility, abortion, still birth, calf death)	Cattle (*n* = 69)			
	Yes	10	14.5	6.2–22.8
	Buffalo (*n* = 20)			
	Yes	2	10.0	0–23.1
What do you do with placental membranes after a cow has given birth?	a) Household consumes	46	61.3	50.3–72.3
	b) Sell	0	0	-
	c) Leave in field	4	5.3	0.2–10.4
	d) Dam consumes	16	21.3	12.0–30.6
	f) I don’t know	8	10.7	3.7–17.7
	a) and c)	1	1.3	0–3.9
		*μ*	**SD**	
When can you diagnose pregnancy? (months)	Cattle (*n* = 67)	3.9	1.2	
	Buffalo (*n* = 18)	5.1	1.9	
Calving to conception interval (months)	Cattle (*n* = 67)	4.5	3.0	
	Buffalo (*n* = 20)	8.5	4.3	

*n*: number of samples, *μ*: mean, SD: standard deviation

### Animal health, nutrition and other management practices

**Fig 3:**
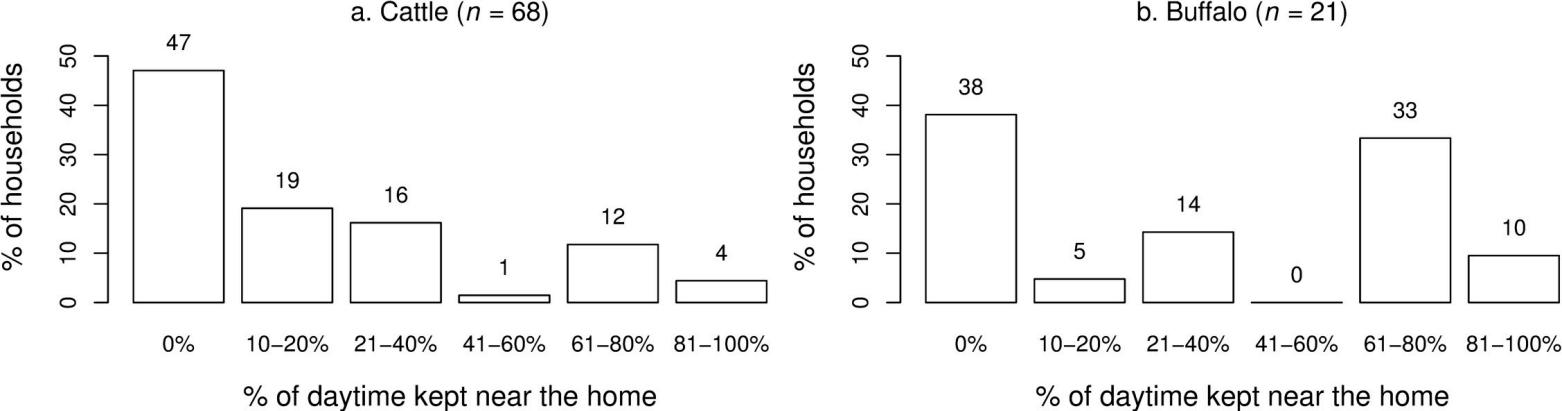
Proportions of smallholder households reporting the amount of daytime (%) that large ruminants were kept near the homestead in 2018, Lao PDR. (a) cattle (b) buffalo.

A summary of animal health, nutritional and other management practices employed by respondents is presented (Table 5). Of the 75 respondents, only 27 (36.0%) reported growing forage. Only 2 farmers (2.7%) reported that their large ruminant water source was mainly from a well or bore, with all remaining farmers reporting natural water reserves. Of the 13 households providing water troughs for large ruminants, 7 households cleaned them once per day, 4 cleaned them once per week and 2 never cleaned them. Of the 69 farmers raising cattle, 33 (47.8%) kept animals housed nightly. Of these, 30/33 (90.9%) reported that their animal house had a roof. For buffalo, 11/21 (52.4%) farmers housed animals nightly and of these farmers, 9/11 (81.8%) reported that their animal house had a roof. A majority of 60/73 (82.2%) of farmers reported that their large ruminants had access to forests for grazing. All 6 buffalo-only households reported that their buffalo had access to forest compared to 44/52 (84.6%) of cattle-only households and 10/15 (66.7%) of cattle-and-buffalo households. Of the 53 households raising cattle only, 19 (35.8%) reported that their cattle came in contact with buffalo. Of the 6 households raising buffalo only, 4 (66.7%) reported that their buffalo came in contact with cattle. Only 12 farmers (16.0%) reported introducing large ruminants to their herds in the last 24 months. Of these 12 farmers, 7 (58.3%) had introduced them from the same village, 4 (33.3%) had introduced them from another village in the same province and 5 (41.7%) had quarantined these animals prior to mixing them with their herds which ranged from 2–120 days. Of the 12 farmers, 7 had introduced large ruminants including pregnant females and calves. Almost all farmers (97.3%) reported vaccinating cattle against FMD and/or Haemorrhagic Septicaemia (HS) in the last 24 months. Of these, 86.8 ± 16.0% of large ruminants were vaccinated. Few farmers (9.3%) slaughtered any livestock on their farm. Buffalo tended to spend more time near the homestead with the largest proportion of households (47%) reporting cattle spent none (0%) of the daytime at home compared to 43% of households reporting buffalo spent more than 60% of the daytime at home (Fig 3).

**Table 5:** Nutritional, biosecurity and management practices in smallholder cattle and buffalo farms in Lao PDR from an epidemiological survey (*n* = 75).

	Category	*n*	%	95% CI
Do you grow forage to feed your cattle?	Yes	27	36.0	25.1–46.9
What is the main source of animal drinking water?	a) Pond/River	73	97.3	93.7–100
	b) Well/Bore	2	2.7	0–6.3
Do you have water troughs for your animals?	Yes	13	17.3	8.7–25.9
Large ruminants are kept in an animal house at night time	Cattle (*n* = 69)	33	47.8	36.0–59.6
	Buffalo (*n* = 21)	11	52.4	31.0–73.8
The animal house has a roof	Cattle (*n* = 33)	30	90.9	81.1–100
	Buffalo (*n* = 11)	9	81.8	59.0–100
Do your large ruminants have access to forest? (*n =* 73)	Yes	60	82.2	73.4–91.0
If you raise **cattle** only, do they come in contact with buffalo? (*n =* 53)	Yes	19	35.8	22.9–48.7
If you raise **buffalo** only, do they come in contact with cattle? (*n* = 6)	Yes	4	66.7	29.0–100
Do you slaughter livestock on the farm?	Yes	7	9.3	2.7–15.9
Have you introduced any large ruminants to your herd in the last 24 months?	Yes	12	16.0	7.7–24.3
If Yes, what was the main place these animals came from? (*n* = 12)	a) same village	7	58.3	30.4–86.2
	b) other village in province	4	33.3	6.6–60.0
If Yes, did you quarantine these animals prior to mixing them with your animals	Yes	5	41.7	13.8–69.6
If Yes, were any of the introduced large ruminants pregnant females or calves	Yes	7	58.3	30.4–86.2
Have you vaccinated your large ruminants against FMD or HS in the last 24 months?	Yes	73	97.3	93.7–100
Is Yes, what % of your animals were vaccinated?		***μ***	**SD**	
		86.8	16.0	

*n;* number of samples, *μ;* mean, SD; standard deviation

### Knowledge of abortifacient and zoonotic disease

Only 31/75 (41.3%) of respondents identified that abortion in cattle can be caused by disease. Fewer farmers (27/75) identified that humans can get diseases from large ruminants and even fewer (18/75) believed that diseases could be transmitted from rats and dogs to large ruminants (Table 6).

**Table 6:** Farmer awareness of abortifacient and zoonotic disease in cattle and buffalo in an epidemiological survey (*n* = 75).

	Category	*n*	%	95% CI
Can abortion in large ruminants be caused by disease?	Yes	31	41.3	30.1–52.4
	No	16	21.3	12.0–30.6
	I don’t know	34	45.3	34.0–56.6
Do you think your family can get diseases from large ruminants?	Yes	27	36.0	25.1–46.9
	No	13	17.3	8.7–25.9
	I don’t know	34	45.3	34.0–56.6
Do you think large ruminants can get disease from dogs or rodents?	Yes	18	24.0	14.3–33.7
	No	13	17.3	8.7–25.9
	I don’t know	44	58.7	47.6–69.8

### Farmer engagement in disease risk practices and their associated factors

#### *N*. *caninum*

**Fig 4:**
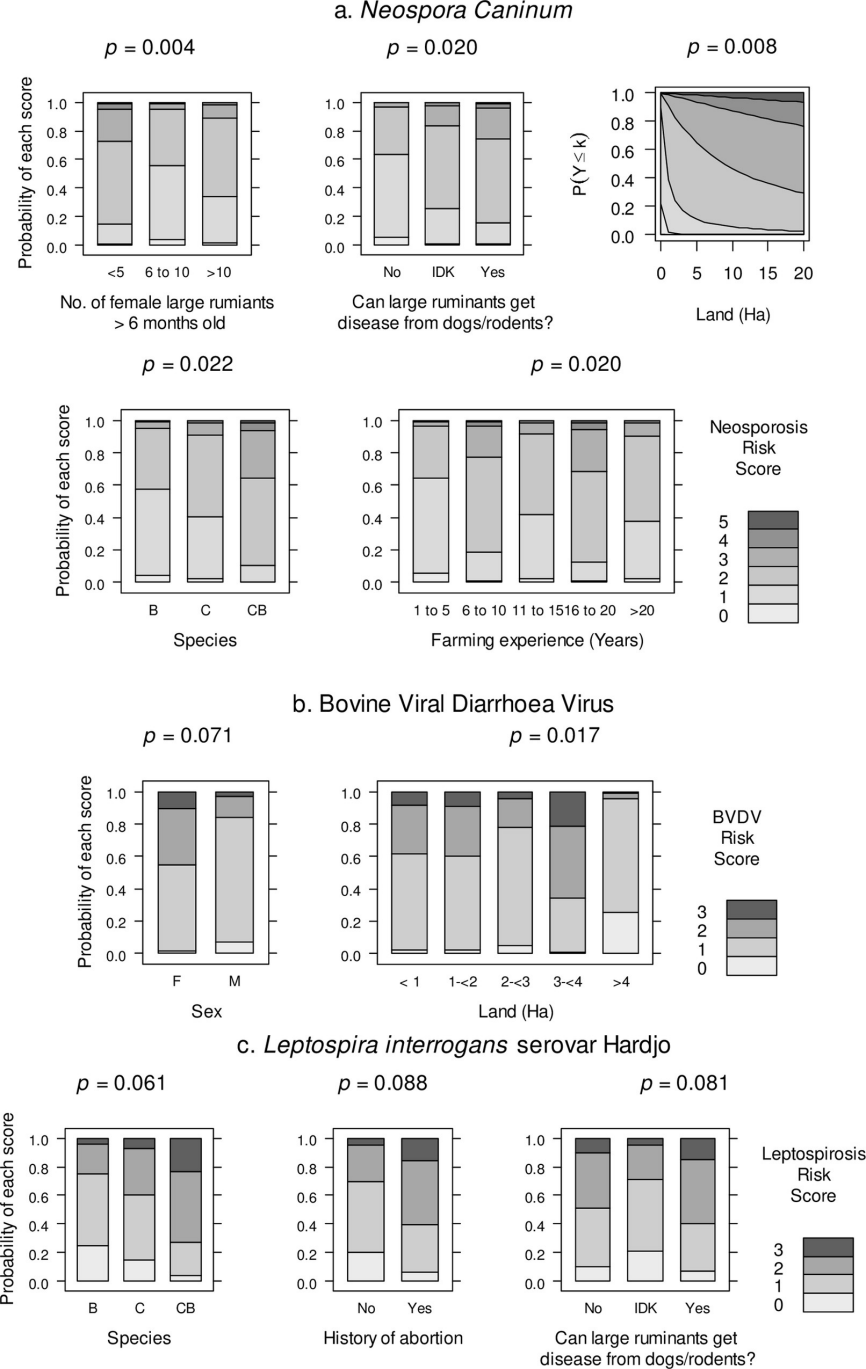
Predicted probability of farmers engaging in risk practices for bovid reproductive diseases from significant predictor variables in final multivariable ordinal regression models in 2018, Lao PDR. (a) Neosporosis, (b) Bovine Viral Diarrhoea virus infection (BVDV) and (c) Leptospirosis. IDK: I don’t know; F: female; M: male, B: buffalo; C: cattle; CB: cattle and buffalo.

Farmers participated in known risk practices for *Neospora caninum* (Table 7) with 58.7% of respondents reporting that dogs consumed aborted foetuses, placental membranes or deceased calves. A further 16% of respondents reported that they had observed dogs and/or rodents defecating or urinating near large ruminant feed sources. A majority of 76.0% of farmers did not isolate calving animals, 18.7% of farmers borrowed farming equipment from neighbours, and 61.3% removed manure from calving areas less than once per week. All but 3 farmers reported participation in at least 1 neosporosis risk practice and the mean risk score was 2.1 ± 1.1 (/5) with a range of 0–5. The final multivariable ordinal logistic model showed that lower risk scores were associated with farmers with fewer female large ruminants under 6 months of age (< 5 head) (*p* = 0.004), with smaller land size (*p* = 0.008), less farming experience (1–5 years) (*p* = 0.020), did not think that large ruminants could get diseases from dogs/rodents (*p* = 0.020), and raised buffalo only (*p* = 0.022) (Fig 4).

**Table 7:** Smallholder participation in known risk practices for contracting reproductive pathogens in large ruminants and their average risk scores.

*Neospora caninum*	*n* (% ± 95 CI)	Bovine viral diarrhoea virus	*n* (% ± 95 CI)	*Leptospira interrogans*	*n* (% ± 95 CI)
Dogs eat aborted foetus/ placenta/ dead calves	44 (58.7 ± 11.1)	Presence of goats	5 (6.7 ± 5.6)	Presence of rodents	7 (9.3 ± 6.6)
Dogs and/or rodents defecating near large ruminant feed	12 (16.0 ± 8.3)	Introduced large ruminants to herd in last 24 months	12 (16.0 ± 8.3)	Dogs and/or rodents defecate or urine near bovid feed	12 (16.0 ± 8.3)
Calving animals not isolated from herd	57 (76.0 ± 9.7)	Cows share bulls	15 (20.3 ± 9.2)	Allow grazing near flooded rice plots	28 (37.8 ± 11.1)
Borrow equipment from neighbours	14 (18.7 ± 8.8)	Common grazing	59 (78.7 ± 9.3)	Presence of pigs	29 (38.7 ± 11.0)
Manure not removed from calving areas weekly	46 (61.3 ± 11.3)			Introduced large ruminants to herd in last 24 months	12 (16.0 ± 8.3)
Mean risk score (/5)	2.1 ± 1.1^a^	Mean risk score (/4)	1.2 ± 0.7^a^	Mean risk score (/5)	1.2 ± 0.9^a^

^a^ mean ± SD

#### BVDV

Farmers participated in known risk practices for BVDV (Table 7) with 6.7% of farmers owning goats, 16.0% introducing large ruminants to their herds in the last 24 months (which included pregnant dams), 20.3% of farmers reporting that their cows shared bulls, and 78.7% of farmers submitted their bovids to common grazing. All but 6 farmers reported participation in at least 1 BVDV risk practice and the mean risk score was 1.2 ± 0.7 (/4) with a range of 0–3. The final multivariable ordinal logistic regression model showed that lower risk scores were associated with farmers owning more available land (> 4 ha) (*p* = 0.017) and were male (*p* = 0.071) (Fig 4).

#### *L*. *interrogans* serovar Hardjo

Farmers participated in risk practices for bovid leptospirosis (Table 7) with 9.3% reporting the presence of rodents at their farm, 16.0% reported that they had observed dogs and/or rodents defecating or urinating near large ruminant feed sources, 37.8% allowed large ruminants to graze around flooded rice plots, 16.0% introduced large ruminants to their herds in the last 24 months, and 38.7% had pigs on their farm. A minority (19/75) of farmers did not report participating in any leptospirosis risk practices and the mean risk score was 1.2 ± 0.9 (/5) with a range of 0–3 (Table 7). The final multivariable ordinal logistic regression model suggested that lower risk scores were associated with farmers with cattle or buffalo (not both) (*p* = 0.061), no history of herd reproductive problems (*p* = 0.088) and farmers who didn’t know whether large ruminants could get diseases from dogs or rodents (*p* = 0.081) (Fig 4).

### Animal level seroprevalence and associated risk factors

**Fig 5:**
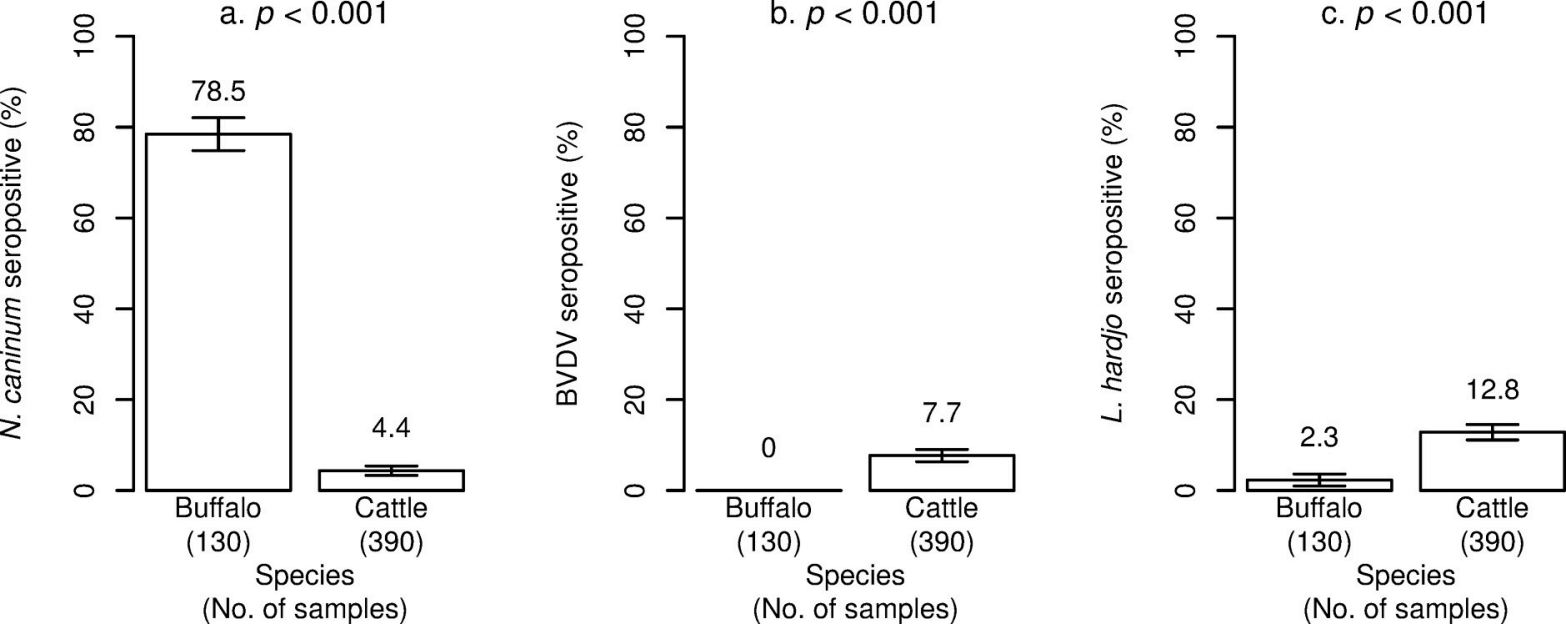
Prevalence of antibodies in cattle and buffalo identified by the enzyme-linked immunosorbent assays in 2018, Lao PDR. **(**a) *Neospora caninum*, (b) bovine viral diarrhoea virus (BVDV) and (c) *Leptospira interrogans* serovar Hardjo (*L*. *hardjo*).

At the S/P cut-off ratio of 21%, *N*. *caninum* seroprevalence was 78.5% (95% CI 71.4–85.6) in buffalo and 4.4% (95% CI 2.4–6.4) in cattle. Antibodies against BVDV were not detected in buffalo but detected in 7.7% (95% CI 3.1–12.3) of cattle samples. Antibodies against *L*. *interrogans* serovar Hardjo were detected in 2.3% (95% CI 0–4.9) of buffalo sera and 12.8% (95% CI 9.5–16.1) of cattle sera. Seroprevalence differed significantly between species for all pathogens (*p* < 0.001) (Fig 5) except *B*. *abortus* where only 1 sample tested positive.

**Fig 6:**
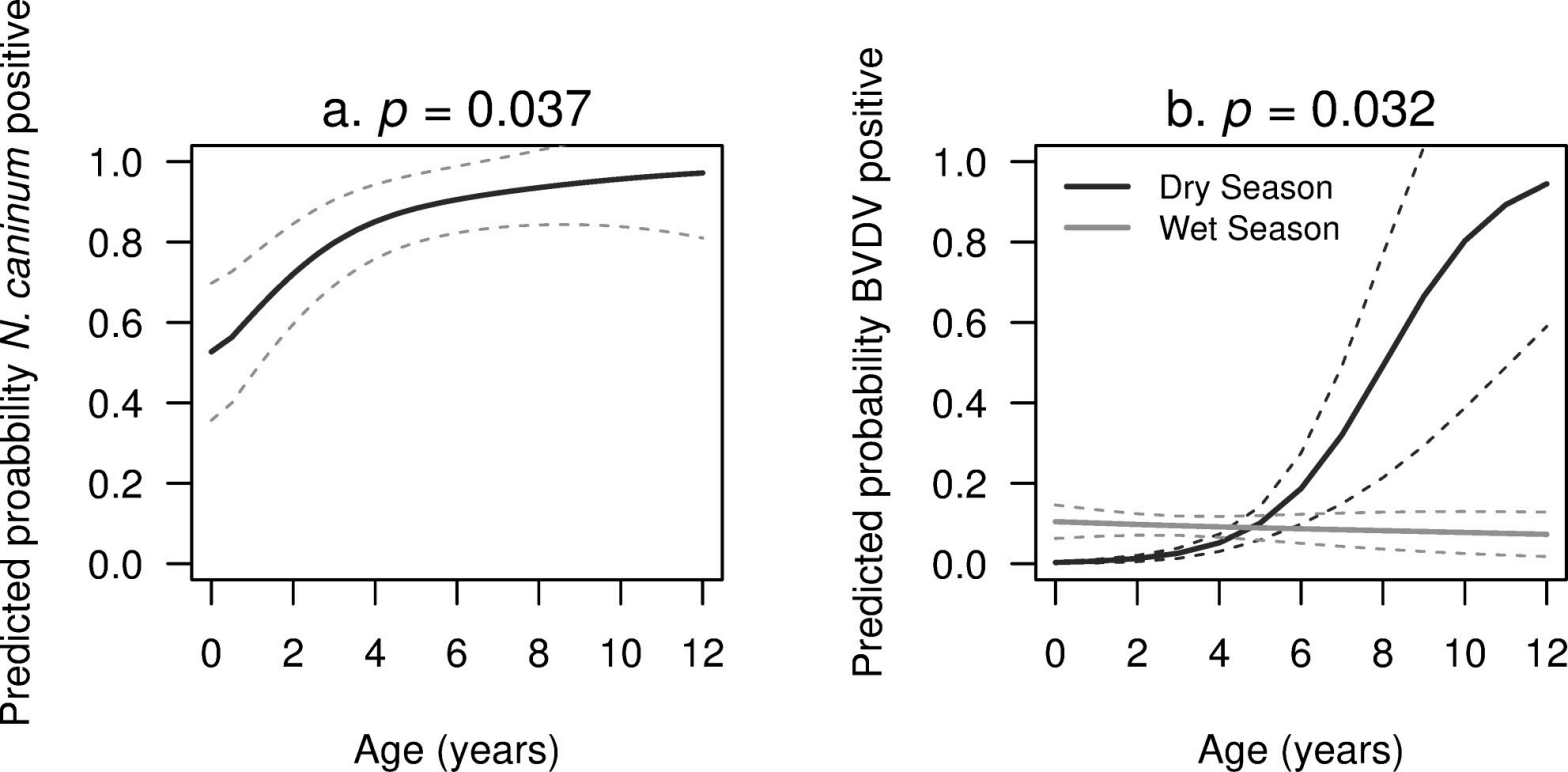
The effect of significant predictors from final multivariable logistic models on the probability that large ruminants were seropositive to infectious pathogens from 2016–2018, Lao PDR. (a) Buffalo age on the probability of being *Neospora caninum* seropositive. (b) The interaction between age and season on the probability of cattle being Bovine Viral Diarrhoea Virus (BVDV) seropositive.

**Fig 7:**
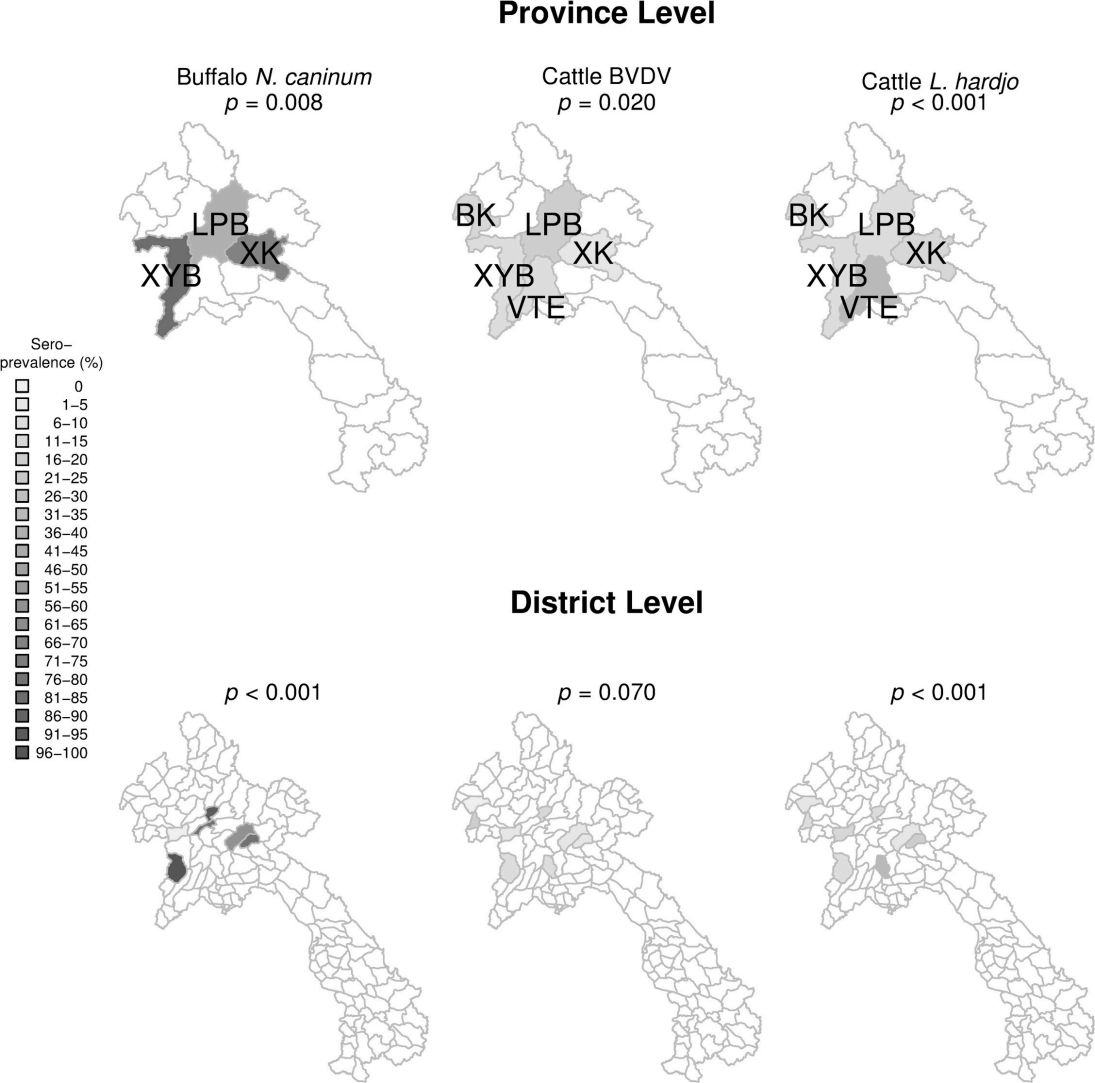
Spatial distribution heat map of buffalo *Neospora caninum*, cattle BVDV and cattle *Leptospira interrogans* serovar Hardjo (*L*. *hardjo*) antibodies in provinces in Lao PDR detected by enzyme-linked immunosorbent assay. Maps and Fisher exact tests to assess variation in sero-prevalence between provinces were generated using R statistical software.

Final multivariable logistic GLMMs for buffalo *N*. *caninum*, cattle *L*. *interrogans* serovar Hardjo and cattle BVDV are presented (Table 8). Buffalo *N*. *caninum* was significantly associated with increasing animal age (*p* = 0.048) where buffalo at birth had a predicted seroprevalence of 52.8 ± 17.0%. For each additional year of age there was a 1.4-fold increase in the odds of being seropositive, increasing the predicted seroprevalence to 97.2 ± 16.2% by age 12 (Fig 6). For *L*. *interrogans* serovar Hardjo in cattle, female cattle had a 2.5-fold increase in the odds of being seropositive compared to males (*p* = 0.034) and samples taken in the wet season had a 2.7-fold increase in the odds of being seropositive compared to the dry season (*p* = 0.077). Cattle had a higher probability of being seropositive as BVDV antibody titres increased (*p* = 0.044). For BVDV in cattle, males had a 3-fold increase in the odds of being seropositive compared to females (*p* = 0.034). Cattle under 4 years of age sampled in the wet season had a higher probability of being seropositive compared to those sampled in the dry season. However, cattle sampled in the dry season had a 2-fold increase in the odds of being seropositive for each yearly increase in age (*p* = 0.032) (Fig 6). Increasing antibodies titres to *N*. *caninum* (*p* = 0.049) and *L*. *interrogans* serovar Hardjo (*p* = 0.065) were associated with increased odds of being BVDV-seropositive. The spatial distribution of these outcome variables is displayed (Fig 7).

**Table 8:** Animal-level risk factors associated with buffalo *Neospora caninum*, cattle *Leptospira interrogans* serovar Hardjo (*L*. *hardjo*) and bovine viral diarrhoea virus (BVDV) seroprevalence in Lao PDR from multivariable logistic generalised linear mixed models.

Predictors	Levels	*b*	SE	OR	95%CI	*p-value*
Buffalo *N*. *caninum*						
Age	-	0.34	0.16	1.41	1.04–1.91	0.037
Cattle *L*. *interrogans* serovar Hardjo						
Sex	Male	0	-	1	-	0.032
	Female	0.93	0.43	2.53	1.08–5.91	
BVDV S/P ratio		0.70	0.35	2.01	1.02–4.0	0.044
Season	Dry	0	-	1	-	0.075
	Wet	1.0	0.54	2.71	0.94–7.75	
Cattle BVDV						
Sex	Female	0	-	1	-	0.018
	Male	1.14	0.48	3.12	1.22–7.99	
Season: age	Dry: Age	0	-	1	-	0.032
	Wet: Age	-0.75	0.35	0.47	0.24–0.93	
*N*. *caninum* OD	-	0.62	0.31	1.87	1.01–3.45	0.049
*L*. *hardjo* OD	-	0.37	0.20	1.45	0.98–2.15	0.066
Season	Dry	0	-	1	-	0.419
	Wet	3.62	1.57	37.27	1.72–809.0	
Age	-	0.72	0.32	2.04	1.10–3.80	0.447

*b*: regression coefficient; SE: standard error; OR: Odds ratio; CI: confidence interval; S/P: sample/positive; OD: optical density

### Herd level seroprevalence and associated risk factors

*N*. *caninum* seroprevalence was higher in buffalo-only herds (*n* = 6) at 83.3% (95% CI 74.6–92), compared to mixed cattle-and-buffalo herds at 18.8% (95% CI 9.7–27.9) (*n* = 16) and cattle-only herds at 10.2% (95% CI ± 3.2–17.2) (*n* = 49). Conversely, herd-level BVDV was highest in cattle-only herds at 14.3% (95% CI 6.2–22.4) followed by cattle-and-buffalo herds at 6.3% (95% CI 0.7–11.9) and absent in buffalo-only herds. Similarly, *L*. *interrogans* serovar Hardjo was absent in buffalo-only but was highest in cattle-and-buffalo herds at 25.0% (95% CI 14.9–35.1), followed closely by cattle-only herds at 24.5% (95% CI 14.5–34.5.0).

Multivariable logistic GLMMs identified factors associated with herd sero-status (Table 9). For *N*. *caninum*, increasing farmer age was associated with reduced odds of a herd being positive (*p* = 0.030). Farmers who reported the presence of rodents on their farms had a 1.7-fold increase in the odds of their herds being positive (*p* = 0.055) and farmers who did not know whether large ruminant feed was contaminated by *N*. *caninum* or rodent excreta had higher odds of being positive compared to farmers who answered yes or no (*p* = 0.092). For herd BVDV sero-status, an increasing proportion of large ruminants vaccinated against FMD/HS was associated with a decreased odds of herd positivity (*p* = 0.005) while an increasing number of female large ruminants was associated with an increased odds of herd positivity (*p* = 0.073). Herds positive for *N*. caninum had a 6.7-fold increase in the odds of being BVDV-positive (*p* = 0.104). For herd *L*. *interrogans* serovar Hardjo sero-status, herds where farmers reported that multiple cows shared bulls (*p* = 0.031) and herds where farmers provided water troughs (*p* = 0.064) had a 14.3- and 20.9-fold increase in the odds of being *L*. *interrogans* serovar Hardjo positive, respectively. Increasing farmer nutrition/reproductive knowledge scores were associated with decreased odds that herds were positive (*p* = 0.046) while increasing farmer experience was associated with increased odds that herds were positive (*p* = 0.108).

**Table 9:** Herd-level risk factors associated with *Neospora caninum*, Bovine viral diarrhoea virus (BVDV) and *Leptospira interrogans* serovar Hardjo seroprevalence in cattle and buffalo in Lao PDR from multivariable logistic generalised linear mixed models.

Predictors	Levels	*b*	SE	OR	95% CI	*p-value*
*N*. *caninum*						
Farmer age		-0.12	0.05	0.89	0.80–0.99	0.030
Presence of rodents	Yes	3.40	1.74	30.05	0.99–914.63	0.055
Feed contaminated by canine or rodent excreta	No	0	-	1	-	0.092
	IDK	2.24	1.28	9.40	0.76–116.91	
	Yes	-1.04	2.14	0.35	0.01–23.4	
*L*. *interrogans* serovar Hardjo						
Multiple cows share a bull	Yes	2.66	1.23	14.31	1.29–158.46	0.031
Farmer knowledge Score (/7)		-1.02	0.51	0.36	0.13–0.98	0.046
Uses water troughs	Yes	3.04	1.67	20.89	0.79–555.77	0.064
Farming experience(years)		0.13	0.08	1.14	0.97–1.34	0.108
BVDV						
FMD/HS Vaccinated (%)		-0.09	0.03	0.91	0.85–0.97	0.005
No. female large ruminants		0.13	0.07	1.14	0.99–1.31	0.073
Herd *N*. *caninum* Ab status	- ve	0	-	1		0.104
	+ve	1.91	1.17	6.74	0.68–66.88	

*b*: regression coefficient; SE: standard error; OR: Odds ratio; CI: confidence interval; IDK: I don’t know; FMD/HS: Foot and Mouth disease or Haemorrhagic Septicaemia; Ab: antibody.

## Discussion

This study builds on preliminary serological evidence of *N*. *caninum*, BVDV and *L*. *interrogans* serovar Hardjo infections in Laos by assaying a larger sample of large ruminant sera and identifying animal- and herd-level risk factors and associations that potentially reduce reproductive performance. *N*. *caninum* seroprevalence of 78.5% in buffalo was significantly higher than co-reared cattle of 3.6% (Fig 3), supporting reports that buffalo are more susceptible to *N*. *caninum* exposure than cattle [9, 13]. Model-based predictions showed that buffalo had a 52.8% chance of being seropositive at birth. This incriminates *in utero* transmission in the epidemiology of neosporosis in buffalo and is consistent with reports that congenital transfer of *N*. *caninum* in buffalos can be a highly efficient route of transmission [9, 46]. Post-natal infection accounted for the remaining exposure as the probability of being seropositive increased 1.4-fold for each additional year of age resulting in mature buffalo having an almost 100% chance of being seropositive (Fig 6). The role of free-roaming, semi-domesticated village dogs in transmission was substantiated by ‘whether rodent or canines had contaminated herd feed with urine or faeces’ being a suggestive predictor of herd-level *N*. *caninum* seroprevalence (Table 9). Farmers answering, ‘I don’t know’ had a 10-fold increase in the odds of their herd being *N*. *caninum* positive relative to farmers who answered ‘no’. This suggests that increasing farmer vigilance probably enables removal of contaminated infectious material whereas farmers who are less watchful do not. The source of canine infection could be through consumption of aborted large ruminant foetuses, placental membranes or dead calves which occurred in 59% of herds. While the role of village dogs is strongly implicated in horizontal bubaline *N*. *caninum* transmission in Laos, future testing of canine serum and faeces may determine the source of canine infection. As rabies is endemic in Laos [47], such studies should involve adequate safety protocols. While the number of buffalo sampled in this study was double the sample size of the preliminary screening, due to the declining numbers of buffalo reducing the availability of samples, a degree of caution should be applied to these results.

Bovid *N*. *caninum* ‘horizontal’ transmission may not be facilitated by canines alone. Multivariable logistic modelling also identified the presence of rodents as a significant risk factor, increasing the probability of herd positivity by 30-fold (Table 9). Rodents have been identified as an intermediate host of *N*. *caninum* in Australia, Italy, The Netherlands, Brazil, Mexico and India [48–50]. While their role in *N*. *caninum* epidemiology is not well understood, a sylvatic life cycle has been hypothesized to exist between wild or domestic canines and rodents [10, 51]. Controlling farm rodents, the main pest of upland rice production, may be an important control measure for reducing the risk of neosporosis in Laos. Recommendations for this include trapping, use of rodenticides (with caution), cats and digging burrows in the rice breeding season [52, 53]. As increasing farmer age was also associated with a decreased probability of herds being seropositive, interventions programs should target younger farmers, although further social research is needed to examine this relationship. Of note, as the experimental unit in herd-level analyses was the household which reduced the sample size, this analysis is considered less robust than animal-level analyses, reflected in several large confidence intervals (Table 9). Nevertheless, these models had sufficient power because they were able to detect significant trends in data.

The pathogen of most concern as a potential zoonosis, *L*. *interrogans* serovar Hardjo, had a 12.8% seroprevalence in cattle; significantly higher than co-reared buffalo at 2.3% (Fig 3) suggesting cattle are at higher risk of exposure than buffalo. Wet season conditions were confirmed as appropriate for the survival of leptospires as animals sampled in this season had a 3-fold increase in the odds of being seropositive compared to those sampled in the dry season (Table 9). Seroprevalence also differed significantly between locations with Vang Vieng district located in Vientiane province having the highest seroprevalence of 33.3%. Geographically, this district has a relatively higher mean annual temperature and annual precipitation [54]. Hence, it is feasible that the higher seroprevalence in cattle compared to buffalo is linked to the higher proportional population density of cattle in central Laos, where the climate may be more conducive to leptospirosis. This may explain why buffalo seroprevalence was lower despite the predilection of buffalo for the swamp habitats usually considered conducive to increased transmission of this pathogen [55]. Future testing with the gold standard, the microscopic agglutination test, is justified to elucidate a fuller array of species-specific serovars and because the diagnostic performance of the prioCHECK ELISA is yet to be fully determined in the target population.

Another aquatic risk factor identified was the practice of using water troughs; associated with a 20-fold increase in the odds of herds being seropositive. Overall, large ruminants mainly received water from ponds and rivers (97.3%). However, the addition of water troughs was most commonly practiced in Vientiane province (21.4% of farmers) and this may contribute to the higher exposure in this location. This reiterates the importance of disinfecting water troughs daily which was practiced by less than half of farmers using troughs. Given the established links to water and the zoonotic potential of leptospirosis, further studies may aim to optimise the use of flooding indicators to predict leptospirosis outbreak risks in Laos, as previously established in neighbouring Cambodia [56].

Venereal transmission of *L*. *interrogans* serovar Hardjo also appears to be a route of infection in Laos. Female cattle had a 2.5-fold increase in the odds of being positive compared to males, and herds where farmers reported multiple cows were serviced by the same bull had a 14-fold increase in the odds of being seropositive (Table 9). It is interpreted that farmers with fewer bulls were more likely to mate their cows with village bulls, facilitating venereal transmission to naïve herds. This is supported by all respondents reporting that they permitted their bovids to unrestricted breeding (97.3%), and further, as sex segregation, castration and herd isolation are not commonly practiced in Laos [21]. Hence, limiting venereal transmission through controlled breeding including artificial insemination (AI) and potentially leptospirosis vaccinations [57] are possible long-term infection control strategies requiring gradual adaptive support. In the short term, improving farmer knowledge may be the best strategy to lower transmission indirectly. This is based on increasing levels of farmer animal health, nutrition and reproductive knowledge being a significant predictor of herd *L*. *interrogans* serovar Harjo sero-status (Table 9). Interestingly, the number of years spent farming large ruminants was not associated with lowered herd exposure. A similar trend was reported in Vietnam where a greater number of years farming dairy cattle was linked to lower reproductive performance [58]. This suggests that the level of large ruminant experience is not necessarily conducive to improved farm practices. Training farmers on the basic concepts of best-practices including provision of adequate nutrition, quarantining introduced animals, removing excess cow and calf manure and vaccinating animals, are necessary interventions to reduce infectious disease risk on smallholder farms.

Finally, BVDV exposure was present in 7.7% of cattle and no buffalo samples, suggesting that cattle are more susceptible to infection than buffalo [59, 60]. The number of females > 6 months of age was used as an indicator of farm size and increased significantly with the probability of herds being BVDV seropositive (Table 9). This supports suggestions that small herd sizes on Lao farms is a protective factor against BVDV transmission and explains why seroprevalence is low despite no active control measures. However, the projected increases in the national herd to satisfy growing regional and local red meat demand [31] will likely result in increased transmission of BVDV unless control measure are put into place.

Based on identified risk factors, BVDV prevention strategies should focus on increasing biosecurity particularly in the dry season. The increasing probability of cattle being BVDV seropositive with increasing age in animals sampled in the dry season (Fig 6) could be linked to the predominance of dry season peak calving that temporarily increases herd size (Fig 1). Additionally, the heightened regional beef demand from post-harvest festivities may increase the circulation of PI and transiently-infected cattle. While all farmers who purchased large ruminants in our study sourced them within their province, because bovids are increasingly entering the Lao market chain from Thailand where the average herd size is larger, BVDV seroprevalence can exceed 50% and PI animals have been identified [61], hence a greater emphasis on biosecurity is needed. Trade-related exposure is further corroborated by the finding that males had a 3-fold increase in the probability of being seropositive compared to females; consistent with farmers trading more males [19]. A strategy to improve dry season biosecurity may be to facilitate forage growing as this was practiced by only 36% of respondents. This will reduce the reliance on common grazing (practiced by 79% of farmers) to avoid contact with the higher volume of introduced and trafficked animals during post-harvest. Addition of interventions including irrigation and fodder storage as silage, can also assist in reducing dependency on common grazing.

Animal-level and herd-level modelling showed potential evidence of BVDVs immunosuppressive potential (Tables 8–9). This suggests that transiently-infected animals may be more susceptible to other abortifacient pathogens and subsequently have an increased risk of abortion [8]. Alternatively, it could reflect farmers with poorer overall biosecurity. Associations to abortion were undetectable in this study except for *L*. *interrogans* serovar Hardjo where participants with known risk factors were significantly linked to a herd history of reproductive problems and a lack of knowledge of reproductive disease (Fig 4). Average reported calving to conception intervals of 4.5 and 8.5 months in cattle and buffalo (Table 4) match current estimates of inter-calving intervals of 14–21 months [3] and confirm that reproductive efficiency is poor in Laos. As increased abdomen (‘stomach’) or udder size remains the main diagnostic sign of pregnancy (97.3%), with farmers only able to detect pregnancy at 3.9–5.1 months, the proportion of reproductive efficiency attributed to pregnancy loss remains unquantifiable. Future studies should aim to benchmark reproductive loss by promoting the absence of behavioural oestrus to detect pregnancy and by encouraging reproductive record keeping. However, more accurate data may require use of rectal palpation or ultrasound scanning techniques for detection of pregnancy.

Human consumption of large ruminant placental membranes and other offal in soup is common and considered a delicacy in Lao cuisine [20]. Whilst this potentially increases the risks of zoonoses if collected without gloves, the practice appears to be a potential protective factor against *N*. *caninum*. This was more commonly practiced in farms with under 2 ha of land while farmers with more than 2 ha of land tended to have placental disposal methods conducive to canine consumption (Fig 1A). This likely explains why increasing farm size was significantly associated with farmer participation in *N*. *caninum* risk practices (Fig 4). Similarly, farmers with larger herds more frequently reported that dogs could consume bovid tissue. Subsequently, farms with both cattle and buffalo were also associated with risk practice participation which was attributed to these farmers having larger herds on average (Fig 2). Farm characteristics can be used to identify farmers more likely to engage in *N*. *caninum* risk factors and this information can be integrated into infection prevention campaigns.

Farmer knowledge also contributed to participation in neosporosis and leptospirosis risk, with farmers who either believed or were not sure whether large ruminants could contract diseases from dogs and rodents having a significantly higher probability of participating in risky practices (Fig 4). Assuming farmer awareness of cross-species transmission is facilitated by personal experience, it is possible that farms engaging in risky practices have experienced reproductive infection. The fact that increased farming experience did not reduce participation in *N*. *caninum* risk factors (Fig 4) and that knowledge of reproductive and zoonotic disease was lacking (Table 6), supports the need for emerging disease awareness extension programs.

There were gender differences in the participation in risky practices for BVDV. Female survey respondents (*n* = 9), half of which were the primary large-ruminant carers, were more likely to participate in risk factors for BVDV compared to males (Fig 4). This finding indicates potential gender inequality in agricultural knowledge, participation and opportunities in Laos. Encouraging greater involvement of women in biosecurity workshops through gender-sensitive approaches is important in addressing infectious livestock diseases in Laos and can have simultaneous benefits on improving national food security [62].

Land size and farm species were significantly associated with farmer participation in risky practices for BVDV and *L*. *interrogans* serovar Hardjo, respectively. Unlike *N*. *caninum*, increasing land size was associated with a reduction in BVDV risk practices (Fig 4). This most likely reflects that farmers with more land (~3.6 ha) did not submit animals to common grazing whereas those with smaller holdings (~1.5 ha) did. As with neosporosis risk, farmers raising both cattle and buffalo species were more likely to engage in risk practices for leptospirosis (Fig 4). This was attributed to these farms more commonly reporting that dogs and/or rodents could defecate near large ruminant feed sources and that they had observed rodents on their farms. This was probably due to less family labour being available to manage farm rodents. Hence, farms with both cattle and buffalo should be targeted for rodent control, despite the presence of rodents not significantly affecting bovid serology in this study.

A contribution of interest from this research was the quantification of various farm management practices in Laos that have previously only been reported anecdotally or as speculation. An aspect not already discussed was that approximately half of cattle and buffalo keepers housed their animals at night. Of these, most had roofs (80–90%) and most farmers (61%) did not remove manure from calving areas weekly. Future studies should explore the impact of the build-up of manure on animal health and potential mitigation strategies including solar exposure to reduce concentrations of faecal-derived pathogens [63]. The finding that buffalo spend more time near the home compared to cattle (Fig 2) supports the hypothesis that increased time spent within a close range of dogs may contribute to higher buffalo infection with *N*. *caninum* [9]. However, as it was not deemed a significant risk factor and because buffalo also had more access to forests, this interaction failed to be substantiated.

The opportunistic use of serum collected for FMD monitoring and from a buffalo dairy herd combined with a retrospective risk factor survey, enabled enhanced understanding of emerging pathogens of bovine reproductive and human zoonotic importance. For *N*. *caninum*, buffalo experience efficient *in utero* transmission followed by horizontal transmission from village dogs and potentially rodents. *L*. *interrogans* is waterborne, with transmission predominating in the wet season, in warmer and wetter locations and surviving in stagnant water troughs. Unrestricted mating may also facilitate venereal transmission between herds, with higher risk in herds with fewer males. BVDV transmission is facilitated by large herds and animal trading in the post-harvest season. Despite abortion surveillance completely lacking on smallholder farms in Laos, the study was able to show that the participation in *L*. *interrogans* serovar Hardjo transmission risk practices is significantly linked to herds with a history of abortion. However, there was no significant association between seropositivity and abortion or individual risk factors. As the emergence of reproductive disease threatens efforts to enhance food security in Laos and beyond, infection transmission preventative strategies should be considered as interventions capable of limiting disease impacts. These potentially include preventing canine consumption of placental and other membranes, controlling rodents, encouraging forage growing, discouraging common grazing, and enhancing general and reproductive disease knowledge, hygiene and biosecurity amongst farmers, including women.

## Supporting information

S1 Text Risk factor survey for bovine and bubaline reproductive diseases in Lao PDR (English). (PDF)

S2 Text Risk factor survey for bovine and bubaline reproductive diseases in Lao PDR (Lao). (PDF)

S1 Dataset Results from commercially-available enzyme-linked immunosorbent assays conducted on cattle and buffalo serum samples collected from Lao PDR from 2016–2018 and associated information. (XLSX)

S2 Dataset Results from an epidemiological survey conducted on smallholder farmers raising cattle and buffalo in Lao PDR in 2018. (XLSX)
