# Supplementary material for: Risk factors for Neospora caninum, bovine viral diarrhoea virus, and Leptospira interrogans serovar Hardjo infection in smallholder cattle and buffalo in Lao PDR
Source: PLoS One. 2019 Aug 8;14(8):e0220335. doi: 10.1371/journal.pone.0220335 (PMC6687104; doi:10.1371/journal.pone.0220335)
Supplement: S1 Text — (PDF) [file pone.0220335.s001.pdf]

THE UNIVERSITY OF SYDNEY

Risk Factor Survey for bovine and bubaline reproductive diseases in Lao PDR

ACIAR AH/2012/068

Objective of study

The aim of this investigation is to determine the seroprevalence of *Neospora caninum,* bovine viral diarrhoea virus (BVDV) and *Leptospira interrogans* serovars Hardjo in Lao PDR. We will then conduct epidemiological survey to determine their associated risk factors in smallholder cattle and buffalo systems.

The objectives of the study are to:

1. Collect ~200 µL of serum from approximately 520 recently collected serum samples from an FMD monitoring program. This program stratified 27 Lao villages by district and provinces and collected from all age groups, sexes and species (buffalo and cattle).

2. Analyse all samples by commercially available enzyme-linked immunosorbent assay (ELISA) kits at the City University of Hong Kong

3. Conduct 50-90 epidemiological surveys on the **primary large ruminant carer** on farms with sampled animals to ascertain household practices and determine risk association to infection

4. Perform univariable and multivariable logistic regression to identify risk factors associated with household-level, village-level and provincial-level seroprevalence

Interviewer name…………………………… Date of interview…………………………………

Latitude………………………………..….…Longitude………………………………………….

1. Household information

Household ID …………………………………. Farmer Name
…………………………………..

Sex: Male ☐ Female ☐ Age
……………………………………..…..

Province …………………….…. District …………………………. Village
……….………………

1. Are you the primary large ruminant carer on this farm?

Yes ☐ No ☐

1.1 If no, who is the primary large ruminant carer on this farm?

a. Wife ☐ b. Husband ☐ c. Son ☐ d. Daughter ☐

f. Other (specify) ☐ …………………………..……………………….…………....

2. Education level of primary large ruminant keeper (Select 1)

a. No formal education ☐ b. Primary school ☐ d. Secondary school ☐

e. College University ☐ e. Other ☐
…………………………….……….……..…

3. No. years' experience of primary keeper raising large ruminants

(Yrs)…….………………..…...

Total area owned (ha) …………………………. No. of land parcels………………………….….

Proximity to nearest town (km)……………… Proximity to major road (km)
…………………..

2. No. of large ruminants on farm 12 months ago

	Cattle			Buffalo		
	Male >6 m	Female >6 m	Calf <6 m	Male >6 m	Female >6 m	Calf <6 m
No. head						
	Main breed (Select 1):			Main breed (Select 1):		

	Local ☐ Cross breed ☐<br>Other ☐ …………………………..	Local ☐ Cross breed ☐<br>Other ☐ …………………………..

1. Were other animals present on the farm 12 months ago: (Select all that apply)

a. Dogs ☐ b. Rodents ☐ c. Cats ☐ d. Goats ☐

e. Pigs ☐ f. Poultry ☐ g. Other ☐

……………………..……..……….

1.2. If Yes to dogs, how many (including puppies)

(No.)………………………..……..…….

3. Animals Introduced in the Last 12 months

1. Have you introduced any large ruminants to your herd in the last 24 months?

Yes ☐ No ☐ If no, skip to section 4

1.1. If Yes, what was the main place these animals came from? (Select all that apply)

a. Same village ☐ specify No. ……………..…

b. Another Village ☐ specify No. ……………..…&

Village……….……..……......……….

c. Another Province ☐ specify No. ……………..…& Province

………..…………..…...……….

d. Another Country ☐ specify No. …………..……& Country

……………..….……………….

e. I don't know ☐ f. Other ☐

……………………………………………………………

1.2. If Yes, did you quarantine these animals prior to mixing them with your animals?

Yes ☐ No. of days…………………………… No ☐ I don't know ☐

1.3. If Yes, were any of the introduced large ruminants pregnant females?

Yes ☐ (No.)………………………………... No ☐ I don't know ☐

1.4. If Yes, were any of the introduced large ruminants calves < 6 months old?

Yes ☐ (No.)………………………………... No ☐ I don't know ☐

4. Farm level breeding practices

1. What is the main method that your cows get pregnant? (Select 1)

a. Completely random mating ☐ b. Farmer selects bull from his own farm ☐ c. Famer selects bulls from another farm ☐ d. Artificial insemination ☐ e. I don't know ☐ f. Other ☐

specify…………..

2. Do your cows use the same bulls for mating?

Yes ☐ No ☐ I don't know ☐

3. How do you detect if your cows are pregnant (Select 1)

a. Increased stomach size ☐ b. Increased size of udder ☐

c. Does not return to oestrus ☐ d. I don't know ☐

e. Other ☐ specify

………………………………………………………………………………….

4. At how many months of pregnancy do you know if the cow is pregnant? (No. of months)

CATTLE: ……………………………….. BUFFALO: ………………………………….…….

5. Have yours cows had any problems with pregnancy in the last 24 months?

CATTLE: Yes ☐ No ☐ If Yes, what are the health problems (Select all that apply):

a. Difficulty getting pregnant ☐ b. Abortion ☐ c. Still birth ☐

d. Calf death ☐ e. I don't know ☐

BUFFALO: Yes ☐ No ☐ If Yes, what are the health problems (Select all that apply):

a. Difficulty getting pregnant ☐ b. Abortion ☐ c. Still birth ☐

d. Calf death ☐ e. I don't know ☐

6. In which month (s) are most of your calves born?

CATTLE (Select all that apply):

Jan ☐ Feb ☐ Mar ☐ Apr ☐ May ☐ Jun ☐ Jul ☐ Aug ☐ Sep ☐ Oct ☐ Nov ☐ Dec ☐

BUFFALO (Select all that apply):

Jan ☐ Feb ☐ Mar ☐ Apr ☐ May ☐ Jun ☐ Jul ☐ Aug ☐ Sep ☐ Oct ☐ Nov ☐ Dec ☐

7. On average, how many months <u>after</u> a cow gives birth until she gets pregnant again

CATTLE: (No.)…………………………………………………………… I don't know ☐

BUFFALO: (No.)………………………………………………………… I don't know ☐

5. Feeding practices

1. Do you grow forage to feed to your cattle?

Yes ☐ No ☐

2. Do you send animals for common grazing?

CATTLE: Yes ☐ No ☐ If Yes, Year round ☐ Seasonal ☐

BUFFALO: Yes ☐ No ☐ If Yes, Year round ☐ Seasonal ☐

3. Do your cattle or buffalo graze around flooded rice plots?

CATTLE: Yes ☐ No ☐ I don't know ☐

BUFFALO: Yes ☐ No ☐ I don't know ☐

4. Have you seen a. dogs or b. rodents or c. both (circle one) defecating or urinating near large ruminant feed (rice straw/pasture/etc.) in last 24 months? Yes ☐ No ☐ I don't know ☐

5. What is the **main source of water** for your large ruminants? (Select 1)

a. Pond ☐ b. River ☐ c. Well ☐ d. Tap ☐ e. Bore ☐

f. Other ☐ Specify ………………………………..………………………………………...

6. Do you have water troughs for your animals?

Yes ☐ No ☐

6.1. If Yes, How often do you clean the water troughs? (Select 1)

Never ☐ Once per day ☐ Once per week ☐ ≥ Once per month ☐

7. Has your farm experienced severe flooding? (not just normal wet season flooding)

Yes ☐ No ☐ I don't know ☐

7.1. If Yes, When was the last time you had flooding (year)? ………………………..

7.2. If Yes, did any household members get sick after the flooding?

Yes ☐ No ☐ I don't know ☐

6. General management

1. Are your large ruminants kept in animal house at night time?

CATTLE: Yes ☐ No ☐

BUFFALO: Yes ☐ No ☐

1.1. If Yes, Does your animal housing area have a roof?

CATTLE: Yes ☐ No ☐

BUFFALO: Yes ☐ No ☐

2. On average, how much time during the day does your large ruminants spend near the home?

CATTLE (Select 1):

a. 0% ☐ b. 10-20% of the day ☐ c. 21-40% of the day ☐

d. 41-60% of the day ☐ e. 61-80 % of the day ☐ f. 81-100% of the day ☐

g. I don't know ☐

BUFFALO (Select 1):

a. 0% ☐ b. 10-20% of the day ☐ c. 21-40% of the day ☐

d. 41-60% of the day ☐ e. 61-80 % of the day ☐ f. 81-100% of the day ☐

g. I don't know ☐

3. Do your large ruminants have access to forest for grazing?

Yes ☐ No ☐ I don't know ☐

4. If you raise cattle only, do your cattle come into contact with buffalo?

Yes ☐ No ☐ I don't know ☐

5. If you raise buffalo only, do your buffalo come into contact with cattle?

Yes ☐ No ☐ I don't know ☐

7. Animal health management practices

1. Do you borrow equipment from other farmers (eg. tractors)?

Yes ☐ No ☐ I don't know ☐

2. Have you vaccinated your large ruminants against FMD or HS in the last 24 months?

Yes ☐ No ☐

2.1. If yes, what % of your animals were vaccinated?

…………………………………….

3. Do you remove manure from animal housing areas less than one time a week?

Yes ☐ No ☐

4. Do you slaughter livestock (cow, buffalo, goat, pig) on the farm?

Yes ☐ No ☐

8. Reproductive management

1. What do you do when cows are about to give birth?

a. Bring them near your house or to a calving area away from other animals ☐

b. Allow to give birth freely in the field or forest ☐

c. Other ☐ specify…………………………………………………………………………

1.1. If in calving area, do you clean calving area after animal gives birth?

Yes ☐ No ☐

2. What do you do with placental membranes? (Select 1)

a. Family consumes ☐ b. Sell ☐ c. Leave in field ☐

e. Cow eats it ☐ f. I don't know ☐

2.1. Is it possible that the dog eats it? Yes ☐ No ☐ I don't know ☐

3. What do you mainly do with aborted foetuses? (Select 1)

a. Family consumes ☐ b. Sell ☐ c. Leave in field ☐ d. I don't know ☐

3.1. Is it possible that the dog eats it? Yes ☐ No ☐ I don't know ☐

4. Have you had any calves died in the last 24 months? Yes ☐ No ☐

4.1. If Yes, what do you mainly do with calves that die? (Select 1)

a. Family consumes ☐ b. Sell ☐ c. Leave in field ☐ d. I don't know ☐

4.1. Is it possible that the dog eats it? Yes ☐ No ☐ I don't know ☐

5.Have you had any of your adults animals (cattle, buffalo, goats, pigs) died in the last 24 months? Yes ☐ No ☐

5.1. If Yes, what do you do with adult animals that die (cattle, buffalo, goats, pigs) (Select 1)

a. Family consumes ☐ b. Sell ☐ c. Leave in field ☐ d. I don't know ☐

5.1. Is it possible that the dog eats it? Yes ☐ No ☐ I don't know ☐

6. Do you drink the milk from a cow who's calf has died or has aborted?

Yes ☐ No ☐

9. Farmer knowledge about reproduction and reproductive disease

1. Can abortion in large ruminants be caused by diseases?

Yes ☐ No ☐ I don't know ☐

2. Do you think your family can get diseases from large ruminants?

Yes ☐ No ☐ I don't know ☐

3. Do you think large ruminants can get diseases from dogs or rodents?

Yes ☐ No ☐ I don't know ☐

10. Basic knowledge from 068 KAP relating to reproduction

1. Giving a vaccination to pregnant cows or buffalos harms them?

True ☐ False ☐ I don't know ☐

2. A pregnant cow or buffalo needs as much as twice as much feed as a cow or buffalo that is not pregnant

True ☐ False ☐ I don't know ☐

3. An adult cow or buffalo needs about 10 kg of fresh grass each day to keep its weight

True ☐ False ☐ I don't know ☐

4. A cow can have its first calve when it is two years old

True ☐ False ☐ I don't know ☐

THE UNIVERSITY OF SYDNEY

5. All adult cows and bulls are good to breed with
True ☐ False ☐ I don't know ☐
6. Bull selection can produce better calves
True ☐ False ☐ I don't know ☐
7. If my cow is being mounted and is very vocal she is ready for breeding
True ☐ False ☐ I don't know ☐

Thank you for your participation!
