# Supplementary material for: Risk factors for Neospora caninum, bovine viral diarrhoea virus, and Leptospira interrogans serovar Hardjo infection in smallholder cattle and buffalo in Lao PDR
Source: PLoS One. 2019 Aug 8;14(8):e0220335. doi: 10.1371/journal.pone.0220335 (PMC6687104; doi:10.1371/journal.pone.0220335)
Supplement: S2 Text — (PDF) [file pone.0220335.s002.pdf]

ແບບຟອມເກັບຂໍ້ມູນ ການສຶກສາປັດໃຈສ່ຽງຂອງການເກີດພະຍາດທີ່ກ່ຽວກັບລະບົບສ ືບພັນ

ໃນງົວຄວາຍ, ສປປ ລາວ (ACIAR AH/2012/068)

ຈຸດປະສົງຂອງການສຶກສາ : ເພື່ອສຶກສາກ່ຽວກັບປັດໃຈສ່ຽງຕ່າງໆທີ່ກ່ຽວກັບການເກີດພະຍາດທີ່ກ່ຽວຂ້ອງກັບລະບົບສືບພັນ ໂດຍສະເພາະແມ່ນເຊື້ອພະຍາດ *N.caninum*, bovine viral diarrhoea virus (BVDV) ແລະ *Leptospira* serovars ທີ່ເກີດໃນງົວຄວາຍພື້ນເມືອງ ສປປ ລາວ.

ຊື່ຜູ້ເຮັດການສຳພາດ…………………………… ວ ດ ປ ທີ່ເຮັດການສຳພາດ…………………………………

ຈຸດພິກັດຂອງບ້ານ (ເສັ້ນແວງ)……………………(ເສັ້ນຂະໜານ)……………………………………….

1. ຂໍ້ມູນສ່ວນຕົວຂອງຊາວກະສິກອນ

ເຮືອນເລກທີ ………………………………… ຊື່ຊາວກະສິອນ …………………………………..
ເພດ: ຊາຍ ☐ ຍິງ ☐ ອາຍຸ (ປີ) ……………………………………..…..
ບ້ານ ………………….…. ເມືອງ …………………………. ແຂວງ ……….………………
1. ທ່ານເປັນຜູ້ດູແລ (ລ້ຽງສັດ) ຫລັກ ຂອງຄອບຄົວບໍ?
ແມ່ນ ☐ ບໍ່ແມ່ນ ☐
1.1 ຖ້າບໍ່ແມ່ນ, ແມ່ນໃຜເປັນຜູ້ລ້ຽງສັດຫລັກຂອງຄອບຄົວທ່ານ?
a. ເມຍ ☐ b. ຜົວ ☐ c. ລູກຊາຍ ☐ d. ລູກສາວ ☐
f. ຜູ້ອື່ນໆ (ກະລຸນາລະລາຍລະອຽດ) ☐ ……………………………..…………………………….……….....
2. ລະດັບການສຶກສາຂອງຜູ້ທີ່ລ້ຽງສັດຫລັກ ຂອງຄອບຄົວທ່ານ (ເລືອກໄດ້ 1 ຄຳຕອບ)
a. ບໍ່ໄດ້ຮຽນໜັງສື ☐ b. ປະຖົມສຶກສາ ☐ d. ມັດທະຍົມສຶກສາ ☐
e. ວິທະຍາໄລ/ມະຫາວິທະຍາໄລ ☐ e. ອື່ນ (ກະລຸນາລະລາຍລະອຽດ) ☐……….….….….…..…
3. ຜູ້ທີ່ລ້ຽງສັດຫລັກ ຂອງຄອບຄົວທ່ານ ມີປະສົບການໃນການລ້ຽງງົວຄວາຍ ຈັກປີແລ້ວ (ປີ)…….…………….…...
ເນື້ອທີ່ການຜະລິດທັງໝົດຂອງຄອບຄົວ (ຮຕ)………………………… ມີດິນຈັກຕອນ……………………….….
ໄລຍະຫ່າງຈາກຕົວເມືອງ (ກມ)………………… ໄລຍະຫ່າງຈາກເສັ້ນທາງຫລວງ (ກມ) …………………..

2. ຈໍານວນງົວຄວາຍທີ່ຄອບຄົວມີຢູ່ ໃນ 12 ເດືອນທີ່ຜ່ານມາ:

	ງົວ			ຄວາຍ		
	ລວມ	ແມ່	ງົວນ້ອຍ <6 ດ	ລວມ	ແມ່	ຄວາຍນ້ອຍ <6 ດ
ໂຕ						
	ສາຍພັນ (ຫມາຍເລືອກ 1 ຄໍາຕອບ):<br>ພື້ນບ້ານ ☐ ພັນຊອດ ☐<br>ອື່ນໆ ☐......................			ສາຍພັນ (ຫມາຍເລືອກ 1ຄໍາຕອບ):<br>ພື້ນບ້ານ ☐ ພັນຊອດ ☐<br>ອື່ນໆ ☐......................		

1. ສັດປະເພດອື່ນໆໃນຄອບຄົວ (ຟາມ) ຂອງທ່ານ ທີ່ມີຢູ່ ໃນໄລຍະ 12 ເດືອນທີ່ຜ່ານ (ເລືອກໄດ້ຫລາຍກວ່າຫນື່ງຄໍາຕອບ)

a. ຫມາ ☐ b. ຫນູ ☐ c. ແມວ ☐ d. ແບ້ ☐

e. ຫມູ ☐ f. ສັດປີກ ☐ g. ອື່ນໆ ☐ ……………………..……..……….

1.2. ຖ້າຄອບຄົວຂອງທ່ານໄດ້ລ້ຽງຫມາ (ລວມທັງລູກຫມາ), ທ່ານມີຫມາທັງຫມົດຈັກໂຕ (ໂຕ)……

3. ຈໍານວນງົວຄວາຍທີ່ຊື້ເຂົ້າມາໃນຝູງໃນຮອບ 12 ເດືອນທີ່ຜ່ານມາ

1. ທ່ານໄດ້ຊືງົວຄວາຍເຂົ້າມາໃນຝູງຂອງທ່ານບ◌ໍໃນຮອບ 24 ເດືອນທີຜ່ານມາ?

ແມ່ນ ☐ ບໍ່ແມ່ນ ☐ ຖ້າຕອບວ່າ **ບໍ່ແມ່ນ** ໃຫ້ຂ້າມໄປ ພາກທີ **4.ການຈັດການດ້ານການຂະຫຍາຍພັນສັດເລີຍ**

1.1. ຖ້າແມ່ນ, ທ່ານຊື້ສັດດັ່ງກ່າວ ມາຈາກໃສເປັນຫລັກ (ເລືອກໄດ້ຫລາຍກວ່າຫນື່ງຄໍາຕອບ)

a. ພາຍໃນບ້ານ ☐ ຈໍານວນສັດທີ່ຊື້ເຂົ້າ. ……………..…

b. ຕ່າງບ້ານ ☐ ຈໍານວນສັດທີ່ຊື້ເຂົ້າ.. ……………..…& ຊື້ຈາກບ້ານ……….……..……......……….

c. ຕ່າງແຂວງ ☐ ຈໍານວນສັດທີ່ຊື້ເຂົ້າ.. ……………..…& ຊື້ຈາກແຂວງ ……….………….…...……….

d. ຕ່າງປະເທດ ☐ ຈໍານວນສັດທີ່ຊື້ເຂົ້າ.. …………..……& ຊື້ຈາກປະເທດ ……………..…….………….

e. ຂ້ອຍບໍ່ຮູ້ແຫລ່ງທີ່ມາ ☐

1.2. ຖ້າແມ່ນ, ທ່ານໄດ້ຂັງແຍກສັດດັ່ງກ່າວ ກ່ອນຈະນໍາເຂົ້າຝູງສັດຂອງທ່ານບໍ?

ແມ່ນ ☐ ໄລຍະເວລາທີ່ຂັງແຍກ (ອັນ)……………………………. ບໍ່ແມ່ນ ☐

1.3. ຖ້າແມ່ນ, ງົວຄວາຍທີ່ທ່ານຊື້ເຂົ້າມານັ້ນ ມີໂຕແມ່ທີ່ຖືພາບໍ?

ແມ່ນ ☐ ຈ◌ໍານວນທີ່ຖືພາ…………………………………... ບໍ່ມີໂຕແມ່ທີ່ຖືພາ ☐

1.4. ຖ້າແມ່ນ, ງົວຄວາຍທີ່ທ່ານຊື້ເຂົ້າມານັ້ນ ມີໂຕງົວຄວາຍນ້ອຍທີ່ອາຍຸຕໍ່າກວ່າ 6 ເດືອນບໍ?

ແມ່ນ ☐ ຈ◌ໍານວນງົວຄວາຍນ້ອຍ……………………ບໍ່ມີງົວຄວາຍນ້ອຍ ທີ່ອາຍຸຕໍ່າກວ່າ 6 ເດືອນ ☐ ຂ້ອຍບໍ່ຮ◌ູ້ ☐

THE UNIVERSITY OF SYDNEY

4. ການຈັດການດ້ານການຂະຫຍາຍພັນສັດ

1. ທ່ານໄດ້ດຳເນີນການຂະຫຍາຍພັນສັດຂອງທ່ານແນວໃດ? (ເລືອກເອົາ 1 ຄຳຕອບ)

a. ປ່ອຍໃຫ້ປະສົມພັນຕາມທຳມະຊາດ ☐ b. ໃຊ້ພໍ່ພັນທີ່ໄດ້ຄັດເລືອກເອົາໃນຝູງ ☐

c. ໃຊ້ພໍ່ພັນທີ່ໄດ້ຄັດເລືອກເອົາຈາກຕ່າງຝູງ ☐ d. ເຮັດການປະສົມພັນທຽມ ☐ e. ຂ້ອຍບໍ່ຮູ້ ☐

2. ທ່ານໃຊ້ງົວຜູ້ໂຕດຽວ (ເກົ່າ) ເພື່ອປະສົມພັນກັບງົວແມ່ຂອງທ່ານ?

ແມ່ນ ☐ ບໍ່ແມ່ນ ☐ ຂ້ອຍບໍ່ຮູ້ ☐

3. ທ່ານ ຮູ້ໄດ້ແນວໃດວ່າ ງົວຄວາຍແມ່ຂອງທ່ານ ກຳລັງຖືພາ (ເລືອກໄດ້ 1 ຄຳຕອບ)

a. ທ້ອງມັນໃຫຍ່ຂຶ້ນ ☐ b. ເຕົ້ານົມມັນໃຫຍ່ຂຶ້ນ ☐ c. ມັນບໍ່ກັບມາຂຶ້ນເພດ ☐

d. ຂ້ອຍບໍ່ຮູ້ ☐ e. ອື່ນໆ ☐ (ລະບຸລາຍລະອຽດ) ……………………………………………………

4. ງົວຄວາຍແມ່ຂອງທ່ານຖືພາ ໄດ້ຈັກເດືອນ ທ່ານຈຶ່ງຮູ້ວ່າ ມັນຖືພາ? (ເດືອນ)

ງົວ: ……………………………….. ຄວາຍ: ……………………………………..…….

5. ໃນຮອບ 24 ເດືອນຜ່ານມາ ງົວຄວາຍແມ່ທີ່ຖືພາຂອງທ່ານ ມີບັນຫາດ້ານສຸຂະພາບຫຍັງບໍ່?

5.1 ງົວແມ່ຖືພາ: ແມ່ນ ☐ ບໍ່ແມ່ນ ☐ **ຖ້າແມ່ນ** ມັນມີບັນຫາຫຍັງ (ເລືອກໄດ້ຫລາຍກວ່າ 1 ຄຳຕອບ):

a. ຖືພາຍາກ ☐ b. ແທ້ງ/ລຸລູກ ☐ c. ງົວນ້ອຍຕາຍໃນທ້ອງ ☐

d. ງົວນ້ອຍຕາຍຫລັງຈາກເກີດມາໄດ້ຫລາຍກວ່າ ຫນຶ່ງວັນ ☐ e. ຂ້ອຍບໍ່ຮູ້ ☐

5.2 ຄວາຍແມ່ຖືພາ : ແມ່ນ ☐ ບໍ່ແມ່ນ ☐ **ຖ້າແມ່ນ** ມັນມີບັນຫາຫຍັງ (ເລືອກໄດ້ຫລາຍກວ່າ 1 ຄຳຕອບ):

a. ຖືພາຍາກ ☐ b. ແທ້ງ/ລຸລູກ ☐ c. ຄວາຍນ້ອຍຕາຍໃນທ້ອງ ☐

d. ຄວາຍນ້ອຍຕາຍຫລັງຈາກເກີດມາໄດ້ຫລາຍກວ່າ ຫນຶ່ງວັນ ☐ e. ຂ້ອຍບໍ່ຮູ້ ☐

6. ລູກງົວຄວາຍນ້ອຍຂອງທ່ານເກີດໃນຊ່ວງເດືອນໃດຂອງປີ?

ງົວ (ເລືອກໄດ້ຫລາຍກວ່າຫນຶ່ງຄຳຕອບ):

Jan ☐ Feb ☐ Mar ☐ Apr ☐ May ☐ Jun ☐ Jul ☐ Aug ☐ Sep ☐ Oct ☐ Nov ☐ Dec ☐

ຄວາຍ (ເລືອກໄດ້ຫລາຍກວ່າຫນຶ່ງຄຳຕອບ):

Jan ☐ Feb ☐ Mar ☐ Apr ☐ May ☐ Jun ☐ Jul ☐ Aug ☐ Sep ☐ Oct ☐ Nov ☐ Dec ☐

7. ສະເລ່ຍແລ້ວ, ດົນປານໃດ ງົວຄວາຍແມ່ຈຶ່ງກັບມາຖືພາອີກ **ຫລັງຈາກມັນເກີດລູກ** (ເດືອນ)

ງົວ: (ເດືອນ)…………………………………………………………… ຂ້ອຍບໍ່ຮູ້ ☐

ຄວາຍ: (ເດືອນ)………………………………………………………… ຂ້ອຍບໍ່ຮູ້ ☐

5. ການຈັດການດ້ານອາຫານສັດ

1. ທ່ານໄດ້ປູກຫຍ້າເພື່ອລ້ຽງງົວຂອງທ່ານບໍ່?

ແມ່ນ ☐ ບໍ່ແມ່ນ ☐

2. ທ່ານໄດ້ປ່ອຍງົວຄວາຍຂອງທ່ານໃຫ້ຫາກິນຢູ່ຕາມເຂດລ້ຽງສັດລວມຂອງບ້ານບໍ່?

ງົວ: ແມ່ນ ☐ ບໍ່ແມ່ນ ☐ ຖ້າແມ່ນ ປ່ອຍຕະຫລອດປີ ☐ ປ່ອຍໃນໄລຍະນອກລະດູການຜະ

ລິດ ☐

ຄວາຍ: ແມ່ນ ☐ ບໍ່ແມ່ນ ☐ ຖ້າແມ່ນ, ປ່ອຍຕະຫລອດປີ ☐ ປ່ອຍໃນໄລຍະນອກລະດູການຜະລິດ ☐

3. ທ່ານໄດ້ປ່ອຍງົວຄວາຍຂອງທ່ານໃຫ້ຫາກິນຢູ່ບ່ອນພື້ນທີ່ງນ້ຳຖ້ວມ ຫລື ທົ່ງນາທີ່ມີເຄີຍມີນ້ຳຖ້ວມບໍ?

ງົວ: ແມ່ນ ☐ ບໍ່ແມ່ນ ☐ ຂ້ອຍບໍ່ຮູ້ ☐

ຄວາຍ: ແມ່ນ ☐ ບໍ່ແມ່ນ ☐ ຂ້ອຍບໍ່ຮູ້ ☐

4. ທ່ານເຄີຍເຫັນຫມາ (a) ຫລື ຫນູ (b) ຫລື ທັງສອງ (c) ຂີ້ ຫລື ຍ່ຽວ ໃສ່ ອາຫານຂອງງົວຄວາຍຂອງທ່ານບໍ (ເຟືອງ, ທົ່ງຫຍ້າອື່ນໆ) ໃນຮອບ 24 ເດືອນຜ່ານມາ? ແມ່ນ ☐ ບໍ່ແມ່ນ ☐ ຂ້ອຍບໍ່ຮູ້ ☐

5. **ແຫລ່ງນ້ຳຫລັກ** ທີ່ງົວຄວາຍຂອງທ່ານກິນ ແມ່ນມາຈາກໃສ? (ເລືອກໄດ້ 1 ຄຳຕອບ)

a. ຫນອງປາ ☐ b. ແມ່ນ້ຳ ☐ c. ນ້ຳສ້າງ/ ນ້ຳບາດານ☐ d. ນ້ຳກ໋ອກ ☐ e. ນ້ຳອອກບໍ່ ☐

f. ອື່ນໆ ☐ ກະລຸນາບອກລາຍລະອຽດ …………………………………..…………………………………………...

6. ທ່ານມີ ຫລື ເຮັດ ຮາງນ້ຳ ເພື່ອເກັບນ້ຳໃຫ້ສັດຂອງທ່ານກິນບໍ?

ແມ່ນ ☐ ບໍ່ແມ່ນ ☐

6.1. ຖ້າ **ແມ່ນ** ທ່າານໄດ້ລ້າງອະນາໄມ ຮາງນ້ຳດັ່ງກ່າວດຸປານໃດ? (ເລືອກໄດ້ 1 ຄຳຕອບ)

ບໍ່ເຄີຍລ້າງ ☐ ຫນຶ່ງຄັ້ງຕໍ່ວັນ ☐ ຫນຶ່ງຄັ້ງຕໍ່ອາທິດ ☐ ≥ ຫນຶ່ງຄັ້ງຕໍ່ເດືອນ ☐

7. ທົ່ງນາ ແລະ ເນື້ອທີ່ການຜະລິດຂອງທ່ານເຄີຍຖືກນ້ຳຖ້ວມຂັງຫນັກເປັນເວລາດົນບໍ? ແມ່ນ ☐ ບໍ່ແມ່ນ ☐ ຂ້ອຍບໍ່ຮູ້ ☐

7.1. ຖ້າແມ່ນ, ຄັ້ງສຸດທ້າຍທີ່ຖືກນ້ຳຖ້ວມຂັງຫນັກເປັນເວລາດົນແມ່ນປີໃດ? ………………………..

7.2. ຖ້າແມ່ນ, ສະມາຊິກໃນຄອບຄົວຂອງທ່ານມີອາການບໍ່ສະບາຍບໍພາຍຫລັງນ້ຳຖ້ວມ? ແມ່ນ☐ ບໍ່ແມ່ນ ☐ ຂ້ອຍບໍ່ຮູ້ ☐

6. ການຈັດພາມທົ່ວໄປ

1. ທ່ານໄດ້ຂັງງົວຄວາຍ ໃນຄອກຂັງສັດ ໃກ້ເຮືອນຂອງທ່ານບໍ?

ງົວ: ແມ່ນ ☐ ບໍ່ແມ່ນ ☐

ຄວາຍ: ແມ່ນ ☐ ບໍ່ແມ່ນ ☐

1.2. ຖ້າແມ່ນ, ຄອກສັດຂອງທ່ານ ໄດ້ມຸງຫລັງຄາບໍ?

ຄອກ ງົວ: ແມ່ນ ☐ ບໍ່ແມ່ນ ☐

ຄອກ ຄວາຍ: ແມ່ນ ☐ ບໍ່ແມ່ນ ☐

2. ສະເລ່ຍແລ້ວ, ງົວຄວາຍຂອງທ່ານໃຊ້ເວລາຫາກິນໃກ້ເຮືອນຂອງທ່ານຫລາຍປານໃດ?

ງົວ: (ເລືອກໄດ້ 1 ຄຳຕອບ)

a. 0% ☐ b. 10-20% ຂອງວັນ ☐ c. 21-40% ຂອງວັນ ☐

d. 41-60% ຂອງວັນ ☐ e. 61-80 % ຂອງວັນ ☐ f. 81-100% ຂອງວັນ ☐ g. ຂ້ອຍບໍ່ຮູ້ ☐

ຄວາຍ: (ເລືອກໄດ້ 1 ຄຳຕອບ)

a. 0% ☐ b. 10-20% ຂອງວັນ ☐ c. 21-40% ຂອງວັນ ☐

d. 41-60% ຂອງວັນ ☐ e. 61-80 % ຂອງວັນ ☐ f. 81-100% ຂອງວັນ ☐ g. ຂ້ອຍບໍ່ຮູ້ ☐

3. ງົວຄວາຍຂອງທ່ານ ສາມາດຍ່າງຫາກິນຫຍ້າຢູ່ໃນປ່າ ຫລື ຖືກປ່ອຍໃຫ້ຫາກິນຫຍ້າຢູ່ໃນປ່າບໍ?

ແມ່ນ ☐ ບໍ່ແມ່ນ ☐ ຂ້ອຍບໍ່ຮູ້ ☐

4. ຖ້າທ່ານລ້ຽງແຕ່ງົວ, ຝູງງົວຂອງທ່ານໄດ້ຫາກິນຮ່ວມກັບຄວາຍຂອງຊາວບ້ານຄົນອື່ນໆບໍ?

ແມ່ນ ☐ ບໍ່ແມ່ນ ☐ ຂ້ອຍບໍ່ຮູ້ ☐

5. ຖ້າທ່ານລ້ຽງແຕ່ຄວາຍ, ຝູງຄວາຍຂອງທ່ານໄດ້ຫາກິນຮ່ວມກັບງົວຂອງຊາວບ້ານຄົນອື່ນໆບໍ?

ແມ່ນ ☐ ບໍ່ແມ່ນ ☐ ຂ້ອຍບໍ່ຮູ້ ☐

THE UNIVERSITY OF SYDNEY

7. ການຮັກສາສຸຂະພາບສັດ

1. ທ່ານໄດ້ຍືມອຸປະກອນການຜະລິດຈາກເພື່ອນບ້ານຄົນອື່ນໆບໍ (ຕົວຢ່າງລົດໄຖນາ, ອື່ນໆ)?

ແມ່ນ ☐ ບໍ່ແມ່ນ ☐

2. ທ່ານໄດ້ສັກຢາກັນພະຍາດປາກເປື່ອຍລົງເລັບ ແລະ ເຕົ້າໂຮມເລືອດໃຫ້ງົວຄວາຍຂອງທ່ານບໍ ໃນຮອບ 24 ເດືອນທີ່ຜ່ານມາ?

ແມ່ນ ☐ ບໍ່ແມ່ນ ☐

2.1. ຖ້າແມ່ນ, ຈັກ ເປີເຊັນ ຂອງຝູງສັດຂອງທ່ານໄດ້ສັກຢາກັນພະຍາດ (%)?

…………………………………….

3. ທ່ານໄດ້ອະນາໄມຂີ້ໃນຄອກງົວຄວາຍຂອງທ່ານຢ່າງຫນ້ອຍຫນຶ່ງຄັ້ງຕໍ່ອາທິດບໍ?

ແມ່ນ ☐ ບໍ່ແມ່ນ ☐

4. ທ່ານໄດ້ຂ້າສັດ (ງົວ, ຄວາຍ, ແບ້, ຫມູ) ໃນເຂດຫລືບ່ອນລ້ຽງສັດ ຂອງທ່ານບໍ?

ແມ່ນ ☐ ບໍ່ແມ່ນ ☐

8. ການຈັດການດ້ານການຂະຫຍາຍພັນສັດ

1. ທ່ານໄດ້ກະກຽມແນວໃດ ໃນເວລາທີ່ງົວຄວາຍແມ່ຂອງທ່ານ ຈະເກີດລູກ?

a. ນຳງົວຄວາຍແມ່ດັ່ງກ່າວມາມັດໃກ້ເຮືອນ ຫລື ບ່ອນສະເພາະສຳຫລັບເກີດລູກ- ໄກຈາກສັດໂຕອື່ນໆ ☐

b. ປະໃຫ້ງົວຄວາຍແມ່ດັ່ງກ່າວອອກລູກໃນທົ່ງຫຍ້າ ແລະ ຢູ່ປ່າຕາມໃຈມັນ ☐

c. ອື່ນໆ ☐ ກະລຸນາບອກລາຍລະອຽດ………………………………………………………………………….

1.1. ໃນກໍລະນີທີ່ທ່ານມີບ່ອນສະເພາະສຳຫລັບເກີດລູກໃຫ້ງົວຄວາຍແມ່ຂອງທ່ານ, ທ່ານໄດ້ລ້າງອະນາໄມພື້ນທີ່ດັ່ງກ່າວຫລັງຈາກສັດເກີດລູກບໍ? ແມ່ນ ☐ ບໍ່ແມ່ນ ☐

2. ທ່ານໄດ້ເຮັດແນວໃດ ກັບນ້ອງງົວ ນ້ອງຄວາຍ? (ເລືອກໄດ້ 1 ຄຳຕອບ)

a. ນຳມາປຸງແຕ່ງເປັນອາຫານ ☐ b. ຂາຍ ☐ c. ປະໄວ້ຢູ່ໃນທົ່ງຫຍ້າຄືເກົ່າ ☐

d. ປ່ອຍໃຫ້ແມ່ງົວຄວາຍກິນ ☐ f. ຂ້ອຍບໍ່ຮູ້ ☐

2.1. ເປັນໄປໄດ້ບໍ ທີ່ຫມາຈະມາກິນນ້ອງງົວນ້ອງຄວາຍທີ່ຖືກຖິ້ມໄວ້? ແມ່ນ ☐ ບໍ່ແມ່ນ ☐ ຂ້ອຍບໍ່ຮູ້ ☐

3. ທ່ານໄດ້ເຮັດແນວໃດ ກັບລູກງົວທີ່ແທ້ງອອກມາ? (ເລືອກໄດ້ 1 ຄຳຕອບ)

a. ນຳມາປຸງແຕ່ງເປັນອາຫານ ☐ b. ຂາຍ ☐ c. ປະໄວ້ຢູ່ໃນທົ່ງຫຍ້າຄືເກົ່າ ☐ d. ຂ້ອຍບໍ່ຮູ້ ☐

3.1. 2.1. ເປັນໄປໄດ້ບໍ ທີ່ຫມາຈະມາກິນລູກງົວຄວາຍທີ່ແທ້ງຕາຍທີ່ຖືກຖິ້ມໄວ້? ແມ່ນ ☐ ບໍ່ແມ່ນ ☐ ຂ້ອຍບໍ່ຮູ້ ☐

4. ທ່ານເຄີຍມີ ກັບງົວຄວາຍນ້ອຍທີ່ຕາຍໂດຍບໍ່ຮູ້ສາເຫດ ໃນຮອບ24ເດືອນຜ່ານມາ? ແມ່ນ ☐ ບໍ່ແມ່ນ ☐

4.1 ຖ້າແມ່ນ, ທ່ານໄດ້ເຮັດແນວໃດກັບ ຊາກງົວຄວາຍນ້ອຍທີ່ຕາຍໂດຍບໍ່ຮູ້ສາເຫດ? (ເລືອກໄດ້ 1 ຄຳຕອບ)

a. ນຳມາປຸງແຕ່ງເປັນອາຫານ ☐ b. ຂາຍ ☐ c. ປະໄວ້ຢູ່ໃນທົ່ງຫຍ້າຄືເກົ່າ ☐ d. ຂ້ອຍບໍ່ຮູ້ ☐

4.1. ເປັນໄປໄດ້ບໍ ທີ່ຫມາຈະມາກິນງົວຄວາຍນ້ອຍທີ່ຕາຍແລະຖືກຖີ້ມໄວ້? ແມ່ນ ☐ ບໍ່ແມ່ນ ☐ ຂ້ອຍບໍ່ຮູ້ ☐

5. ທ່ານເຄີຍມີ ສັດໃຫຍ່ເຕັມໄວ້ (ງົວ, ຄວາຍ, ແບ້, ຫມູ) ຕາຍໂດຍບໍ່ຮູ້ສາເຫດໃນຮອບ24ເດືອນຜ່ານມາ? ແມ່ນ ☐ ບໍ່ແມ່ນ☐

5.1 ຖ້າແມ່ນ, ທ່ານໄດ້ເຮັດແນວໃດຈາກສັດ ໃຫຍ່ເຕັມໄວ້(ງົວ,ຄວາຍ,ແບ້,ຫມູ) ທີ່ຕາຍໂດຍບໍ່ຮູ້ສາເຫດ?(ເລືອກໄດ້1ຄໍາຕອບ)

a. ນໍາມາປຸງແຕ່ງເປັນອາຫານ ☐ b. ຂາຍ ☐ c. ປະໄວ້ຢູ່ໃນທົ່ງຫຍ້າຄືເກົ່າ ☐ d. ຂ້ອຍບໍ່ຮູ້ ☐

5.1. ເປັນໄປໄດ້ບໍ ທີ່ຫມາຈະມາກິນຈາກສັດທີ່ຕາຍທີ່ຖືກຖີ້ມໄວ້? ແມ່ນ ☐ ບໍ່ແມ່ນ ☐ ຂ້ອຍບໍ່ຮູ້ ☐

6. ທ່ານໄດ້ກິນນໍ້ານົມ ຈາກງົວຄວາຍແມ່ ທີ່ລູກມັນຕາຍ ຫລື ແທ້ງລູກ?

ແມ່ນ ☐ ບໍ່ແມ່ນ ☐

9. ຄວາມຮູ້ດ້ານການຂະຫຍາຍພັນສັດ ແລະ ພະຍາດທີ່ກ່ຽວກັບລະບົບສືບພັນ

1. ງົວຄວາຍແມ່ສາມາດແທ້ງລູກໄດ້ບໍ ຖ້າຫາກມັນຕິດພະຍາດ?

ແມ່ນ ☐ ບໍ່ແມ່ນ ☐ ຂ້ອຍບໍ່ຮູ້ ☐

2. ທ່ານຄິດວ່າ ທ່ານເອງ ແລະ ສະມາຊິກໃນຄອບຄົວຂອງທ່ານ ສາມາດຕິດພະຍາດຈາກງົວຄວາຍທີ່ຕິດເຊື້ອພະຍາດໄດ້ບໍ?

ແມ່ນ ☐ ບໍ່ແມ່ນ ☐ ຂ້ອຍບໍ່ຮູ້ ☐

3. ທ່ານຄິດວ່າງົວຄວາຍສາມາດຕິດເຊື້ອພະຍາດຈາກ ຫມາ ຫລື ຫມູ ທີ່ຕິດພະຍາດໄດ້ບໍ?

ແມ່ນ ☐ ບໍ່ແມ່ນ ☐ ຂ້ອຍບໍ່ຮູ້ ☐

10. ຄໍາຖາມກ່ຽວກັບການຂະຫຍາຍພັນສັດ ທີ່ຄັດມາຈາກ ແບບຟອມດ KAP ຂອງໂຄງການ 068

1. ການສັກຢາກັນພະຍາດໃຫ້ແກ່ງົວຄວາຍແມ່ທີ່ຖືພາຈະເປັນອັນຕະລາຍໃຫ້ແກ່ມັນບໍ?

ແມ່ນ ☐ ບໍ່ແມ່ນ ☐ ຂ້ອຍບໍ່ຮູ້ ☐

2. ງົວຄວາຍແມ່ທີ່ກໍາລັງຖືພາ ຄວນຈະໄດ້ຮັບອາຫານໃນຈໍານວນສອງເທົ່າຂອງງົວຄວາຍແມ່ທີ່ບໍ່ໄດ້ຖືພາບໍ

ແມ່ນ ☐ ບໍ່ແມ່ນ ☐ ຂ້ອຍບໍ່ຮູ້ ☐

3. ງົວຄວາຍໃຫຍ່ເຕັມໄວ ຄວນຈະໄດ້ກິນຫຍ້າສົດຈໍານວນ10ກກ ເພື່ອມັນຈະຍັງຄົງຮັກສານໍ້າຫນັກຂອງມັນໃຫ້ຄືເກົ່າ

ແມ່ນ ☐ ບໍ່ແມ່ນ ☐ ຂ້ອຍບໍ່ຮູ້ ☐

4. ທ່ານຄິດວ່າ ງົວແມ່ສາມາດເກີດລູກໂຕທໍາອິດເມື່ອມັນມີອາຍຸໄດ້ສອງປີໄດ້ບໍ

ແມ່ນ ☐ ບໍ່ແມ່ນ ☐ ຂ້ອຍບໍ່ຮູ້ ☐

5. ທ່ານຄິດວ່າ ງົວຜູ້ ແລະ ງົວແມ່ທຸກໆໂຕສາມາດຄັດເລືອກລ້ຽງໄວ້ເປັນພໍ່ແມ່ພັນໄດ້ຫມົດໄດ້ບໍ

ແມ່ນ ☐ ບໍ່ແມ່ນ ☐ ຂ້ອຍບໍ່ຮູ້ ☐

6. ທ່ານຄິດວ່າການຄັດເລືອກງົວຜູ້ທີ່ມີລັກສະນະທີ່ດີເພື່ອມາເຮັດເປັນພໍ່ພັນ
ຈະສາມາດຜະລິດລູກງົວນ້ອຍທີ່ມີຮູບຮ່າງທີ່ດີແມ່ນບໍ

ແມ່ນ ☐ ບໍ່ແມ່ນ ☐ ຂ້ອຍບໍ່ຮູ້ ☐

7. ທ່ານຄິດວ່າ ຖ້າງົວແມ່ສົ່ງສຽງຮ້ອງຄາງ ແລະ ດົມງົວແມ່ໂຕອື່ນໆ ສະແດງວ່າມັນ**<u>ພ້ອມ</u>**ທີ່ຈະປະສົມພັນແມ່ນບໍ

ແມ່ນ ☐ ບໍ່ແມ່ນ ☐ ຂ້ອຍບໍ່ຮູ້ ☐

ຂອບໃຈທີ່ທ່ານສະຫລະເວລາເຂົ້າຮ່ວມການເກັບຂໍ້ມູນໃນຄັ້ງນີ້!
